# Computational Investigation of Homologous Recombination DNA Repair Deficiency in Sporadic Breast Cancer

**DOI:** 10.1038/s41598-017-16138-2

**Published:** 2017-11-16

**Authors:** Yue Wang, Matthew H. Ung, Sharon Cantor, Chao Cheng

**Affiliations:** 1 School of Electronic Information and Communications at Huazhong University of Science and Technology, Wuhan, Hubei 430074 China; 2 Department of Molecular and Systems Biology, Geisel School of Medicine at Dartmouth College, Hanover, NH 03755 USA; 3 Department of Molecular, Cell and Cancer Biology, University of Massachusetts Medical School, Worcester, MA 01605 USA; 4 Norris Cotton Cancer Center, Geisel School of Medicine at Dartmouth College, Lebanon, NH 03766 USA; 5 Department of Biomedical Data Sciences, Geisel School of Medicine at Dartmouth College, Lebanon, NH 03766 USA

## Abstract

BRCAness has important implications in the management and treatment of patients with breast and ovarian cancer. In this study, we propose a computational framework to measure the BRCAness of breast and ovarian tumor samples based on their gene expression profiles. We define a characteristic profile for BRCAness by comparing gene expression differences between *BRCA1*/2 mutant familial tumors and sporadic breast cancer tumors while adjusting for relevant clinical factors. With this BRCAness profile, our framework calculates sample-specific BRCA scores, which indicates homologous recombination (HR)-mediated DNA repair pathway activity of samples. We found that in sporadic breast cancer high BRCAness score is associated with aberrant copy number of HR genes rather than somatic mutation and other genomic features. Moreover, we observed significant correlations of BRCA score with genome instability and neoadjuvant chemotherapy. More importantly, BRCA score provides significant prognostic value in both breast and ovarian cancers after considering established clinical variables. In summary, the inferred BRCAness from our framework can be used as a robust biomarker for the prediction of prognosis and treatment response in breast and ovarian cancers.

## Introduction

Breast cancer is the most common type of cancer in female patients, with one out of eight women developing breast cancer in their lifetime^1^. Many factors have been found to be associated with the increased risk of this disease including family history^2^. Familial breast cancer represents a minor percentage of all breast cancer cases and can occur in patients with one or more closely related family members diagnosed with breast, ovarian, or related cancer^2,3^. Approximately 25% of familial breast cancer cases may be attributed to germline mutations in two major breast cancer susceptibility genes, *BRCA1* ^4^ and *BRCA*2^5^. Germline mutations in *BRCA1* and *BRCA*2 genes exhibit high penetrance and confer a 60–80% and 40–85% lifetime risk of developing breast cancer, respectively^6^. In ovarian cancer, *BRCA1* and *BRCA*2 germline mutations will confer a risk of 40–60% and 30%, respectively^7^. Other than germline mutations in these two genes, many other genetic mutations or variants can contribute to familial breast cancer including germline mutations with high (*e*.*g*., *TP53* and *PTEN*) and low penetrance (*e*.*g*., *ATM* and *BRIP1*), as well as low-penetrance genetic variants (*e*.*g*., single-nucleotide polymorphisms (SNPs))^8^.

BRCA1 and BRCA2 are essential regulators of the homologous recombination (HR) pathway that are involved in the repair of double-stranded DNA breaks^9,10^. HR-dependent DNA repair restores damaged DNA sequences to its original state without introducing DNA mutations. When this pathway is inactivated, *e*.*g*., as a result of *BRCA1*/2 mutations, alternative DNA repair pathways such as non-homologous end joining (NHEJ) become the major pathways utilized for repairing double-strand DNA breaks. These alternative repair mechanisms are error-prone and lead to rapid accumulation of somatic mutations which increase the risk of carcinogenesis^11,12^. Interestingly, somatic mutations in *BRCA1*/2 are rarely observed in sporadic breast cancers^13,14^. However, defects in HR-dependent DNA repair can arise through other mechanisms, resulting in a “BRCA-like” phenotype. For this reason, the concept of “BRCAness” has been introduced to describe this shared phenotype between sporadic cancers and familial cancers with *BRCA1*/2 mutations^15,16^.

BRCAness has important implications in the management and treatment of patients with sporadic breast and ovarian cancer^15–18^. It has been shown that tumors with defects in the HR-dependent DNA repair pathway are hypersensitive to alkylating, platinum-based chemotherapies that generate DNA interstrand crosslinks and induce double-stranded DNA breaks during crosslink removal^19,20^. However, tumors with deficient BRCA1 activity are not sensitive to mitotic spindle poisons such as the taxanes and vincristine^21^. This is because that spindle disruption caused by these agents can induce apoptotic cell death in BRCA-proficient but not in BRCA-deficient tumors. It has been shown that downregulation of *BRCA1* gene expression in ovarian cancer cell lines increases their sensitivity to platinum treatment but leads to resistance to antimicrotubule agents^22,23^. In addition, tumors with deficient *BRCA1*/2 are also sensitive to poly (ADP-ribose) polymerase (PARP) inhibitors as a result of synthetic lethality^24,25^. In particular, PARP is involved in another DNA repair pathway called base excision repair (BER) that repair single-strand DNA breaks^26^. Inhibition of PARP results in accumulation of DNA single-strand breaks, replication fork collapse and double-strand DNA repair that are lethal in tumors with deficient HR pathway.

The hallmarks of BRCAness are elevated genomic instability and deficient HR pathway activity. Accordingly, there are two strategies to determine the BRCAness of sporadic breast tumor samples. The first strategy is to classify BRCA-like and non-BRCA-like samples based on copy number variation (CNV) data. Classification models have been constructed by selecting genomic regions with differential CNV between familial (with *BRCA1*/2 germline mutations) and sporadic breast cancer samples, and then applying these models to assess BRCAness in sporadic samples^27^. These methods assume that there exist ‘hot’ genomic regions with recurrent CNV shared by the majority of BRCA-deficient familial and BRCA-like sporadic samples. However, this assumption may not hold when considering the high heterogeneity of sporadic tumors. The second strategy is to define gene signatures that characterize HR deficiency and apply them to identify HR-deficient sporadic tumor samples. Konstantinopoulos *et al*.^28^ defined a BRCAness gene signature by comparing transcriptomic profiles between *BRCA1/*2 mutant and sporadic ovarian tumor samples, and applied it to classify sporadic ovarian tumors. Their approach demonstrated that samples with high levels of BRCAness were associated with improved survival. A related approach involves generating BRCAness gene expression profiles from breast cancer cell line with RNA-mediated inactivation of HR pathway genes (*e*.*g*. *BRCA1*, *RAD51* and *BRIT1*)^29^. However, our previous study using this approach showed that the resulting gene expression profiles from these knockdown cell line were more likely to reflect the reduced proliferation of cells rather than HR deficiency^30^.

In this study, we propose a robust statistical framework to characterize a BRCAness gene expression profile to interrogate BRCA activity in sporadic breast cancer samples. The BRCAness profile is generated by comparing gene expression between *BRCA1*/2 mutant familial tumors with sporadic breast tumors. In addition, this statistical model used to define the profile takes into account clinical factors that could explain differences in gene expression, thus effectively isolating the expression changes most likely to be induced by varying levels of BRCAness. This characteristic BRCAness profile utilizes all genes rather than a gene signature composed of a selected group of genes to achieve robust statistical performance. Application of this framework to The Cancer Genome Atlas (TCGA) breast cancer omics data revealed high heterogeneity of BRCAness mechanisms in sporadic breast cancer. Our results indicate that in sporadic breast cancer, copy number variation (CNV), especially deletions, contributes more to the inactivation of HR pathway compared to somatic mutations, DNA methylation, and expression changes in BRCA-like samples. Other than *BRCA1*/2, CNV in other HR genes play a more important role to reduce HR pathway activity in sporadic breast tumors. We also identified several other HR pathway genes with CNV associated with BRCAness, suggesting that other members of the axis may contribute more to HR pathway activity. Moreover, the inferred BRCA score is predictive of clinical outcomes of patients with breast cancer, providing a potential prognostic marker. In addition, this BRCAness profile, although being defined for breast cancer, is also applicable to ovarian cancer.

## Results

### Schematic overview of our study

**Figure 1:**
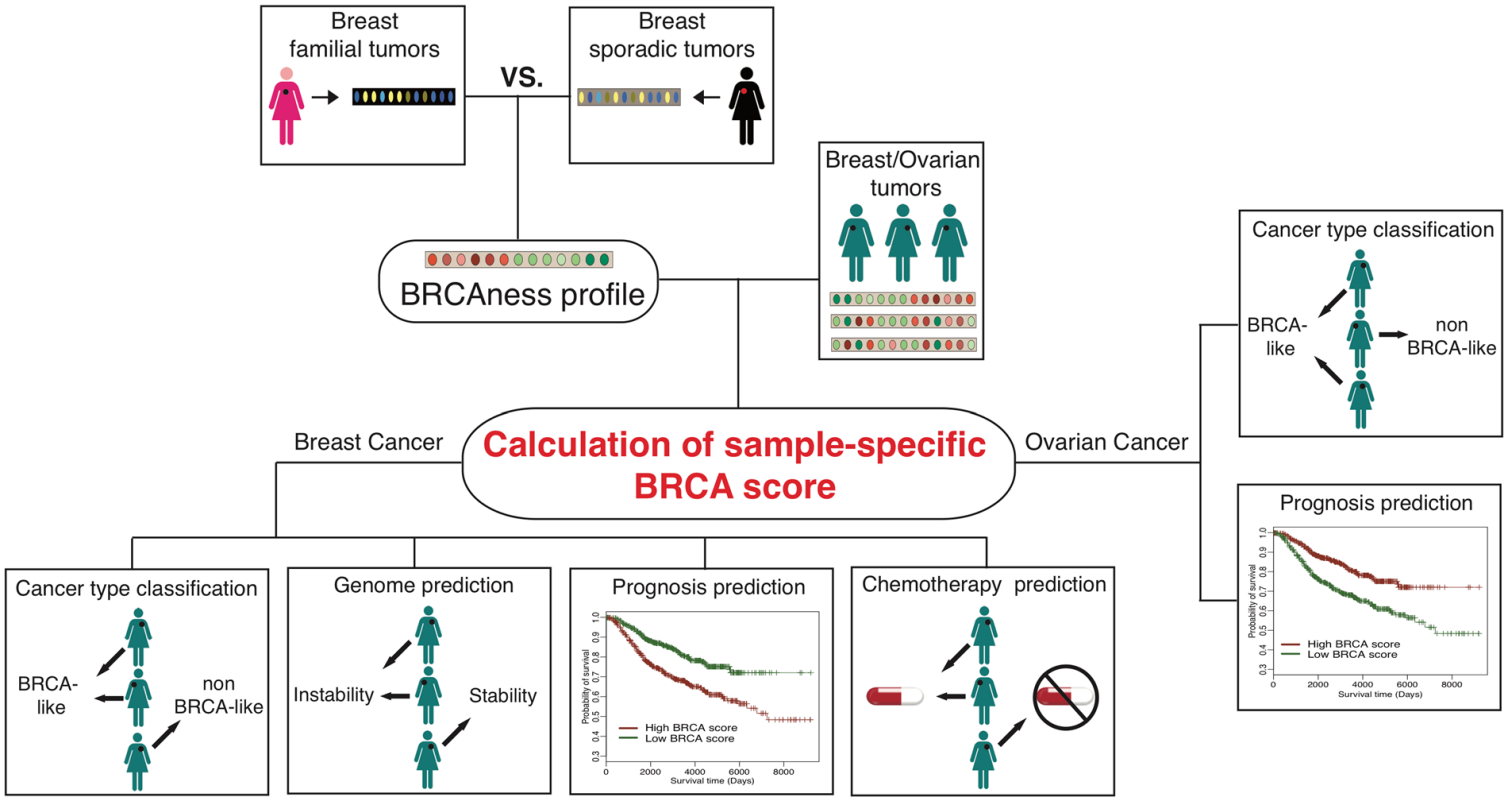
Overview of our computational framework. In short, we first compared the gene expression profiles between familial and sporadic breast tumors considering related clinical factors (*e*.*g*. age, grade, tumor size, ER status and Her2 status) to generate a BRCAness profile. Second, by integrating gene expressions of given breast cancer samples, we calculated sample-specific BRCA scores. The scores inferred the BRCAness of patients with the higher score the higher likelihood to be BRCAness. Then, we showed that the BRCA score classifies familial from sporadic breast tumors, correlates with genomic instability, predicts patients’ survival and predicts chemotherapy response. Lastly, we further applied the BRCAness profile defined with breast cancer profiles to ovarian cancer. The corresponding BRCA scores also can classify familial from sporadic samples and predict prognosis.

Our computational framework starts by comparing familial breast tumors carrying either *BRCA1* or *BRCA*2 mutations with sporadic cancer samples to generate a BRCAness characteristic profile (Fig. 1). This comparison adjusts for several clinical factors including age, grade, tumor size, estrogen receptor (ER) status, and Human Epidermal Growth Factor Receptor 2 (HER2) status to ensure that the BRCAness characteristic profile reflects *BRCA1*/2 mutation status rather than differences in the distribution of these clinical variables across patients. Given a tumor gene expression dataset, this BRCAness profile calculates a sample-specific BRCA score for each individual patient in the dataset by using the BASE algorithm, which measures the similarity between a patient’s tumor expression profile and the BRCAness profile^31^. Patients with higher BRCA scores are more similar to have *BRCA1/*2 germline mutations in terms of their expression profiles, and thereby more likely to have defective HR pathway activity.

### Defining characteristic weight profiles that encode BRCAness

We utilized the data generated by Larsen *et al*.^32^ to define three BRCAness characteristic profiles (denoted as *BRCA1*-, *BRCA*2- and *BRCA1*/2-based profiles) by respectively comparing *BRCA1-*, *BRCA*2*-* or *BRCA1*/2-mutant (both *BRCA1* and *BRCA*2) familial samples with sporadic samples. In a BRCAness profile, each gene was assigned a weight based on the difference in expression it exhibits between *BRCA1*/2-mutated familial from sporadic breast cancer samples. Namely, genes with high weights were significantly up- or down-regulated in *BRCA1*/2-mutated samples after adjusting for several clinical variables that may be potential confounders. We selected 300 genes with the highest positive (up-regulated in *BRCA1*/2-mutant) and 300 genes with the highest negative (down-regulated in *BRCA1*/2-mutant) weights in the BRCAness profile and identified the enriched biological pathways. Our results indicate that cell cycle associated and DNA replication pathways are significantly enriched in the up-regulated genes (Supplementary Table S1). In addition, the same analyses were performed using top weighted genes in BRCAness profiles defined based on *BRCA1* versus sporadic and *BRCA*2 versus sporadic profiles and obtained similar results. These results are consistent with the known functions of BRCA1 and BRCA2 (Supplementary Table S1).

### Calculated BRCA score discriminates familial from sporadic breast tumors

**Figure 2:**
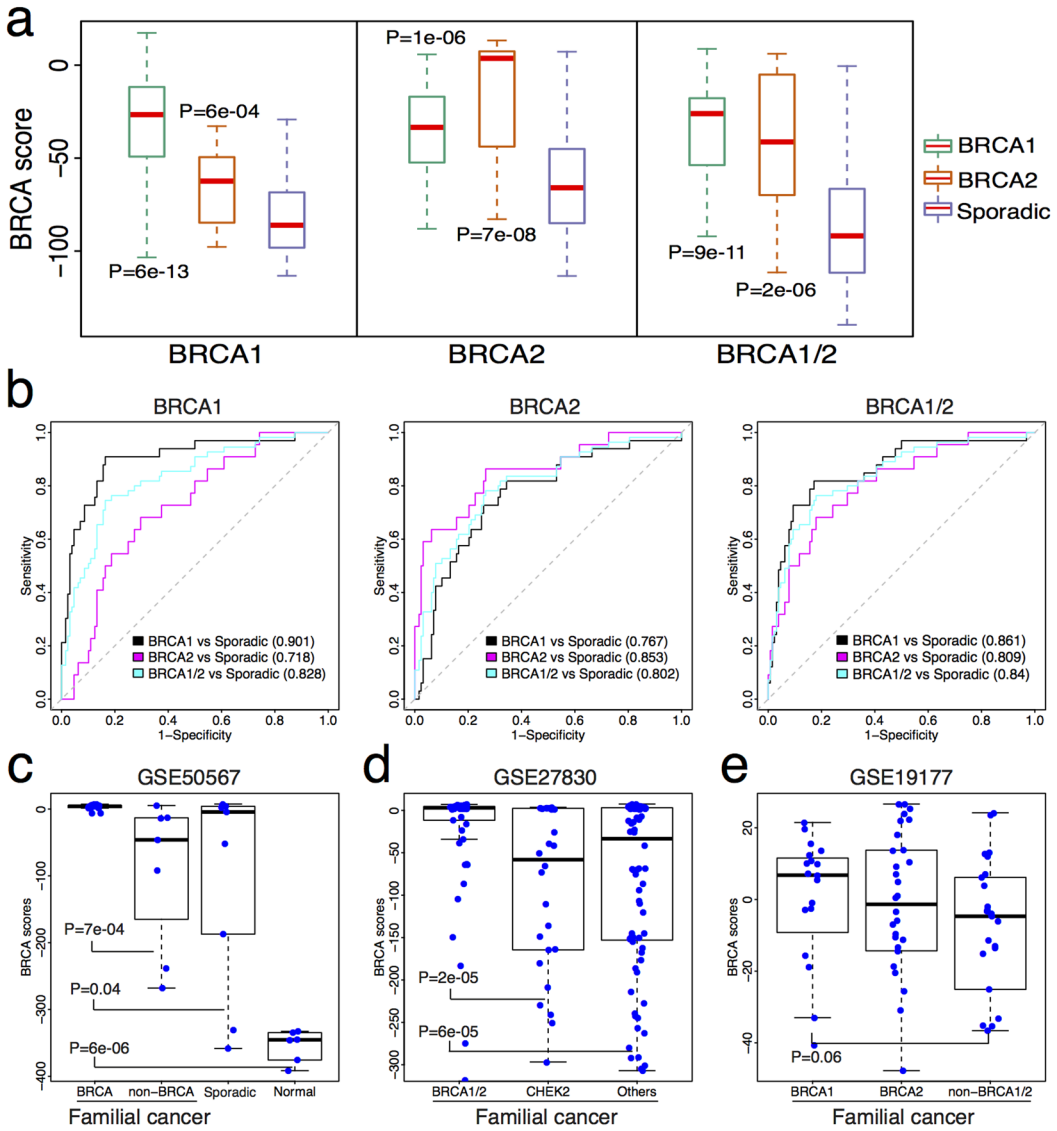
BRCA score classifies familial from sporadic breast cancer patients. (**a**) Boxplot for comparisons of BRCA scores in germline mutate-*BRCA1* (green box), germline mutate-*BRCA2* (tawny box) and sporadic (blue box) breast cancer samples using gene expression of germline mutate-*BRCA1* (left), germline mutate-*BRCA*2 (middle), germline mutate-*BRCA1*/2 (both *BRCA1* and *BRCA2*, right) as reference to compare with those of sporadic ones, respectively. Mann-Whitney Wilcoxon test P-values were calculated to show the differences of BRCA scores between germline mutate-*BRCA1* and sporadic breast cancer samples, germline mutate-*BRCA*2 and sporadic breast cancer samples. (**b**) ROC curves for the accuracy of classifying familial from sporadic breast cancer patients using BRCA scores calculated by comparing gene expression of germline mutate-*BRCA1* (left), germline mutate-*BRCA2* (middle), germline mutate-*BRCA1*/*2* (right) as reference to sporadic ones, respectively. Black curve: comparison of patients with germline *BRCA1* mutations to sporadic ones. Magenta curve: comparison of patients with germline *BRCA2* mutations to sporadic ones. Cyan curve: comparison of patients with germline *BRCA1* and *BRCA2* mutations to sporadic ones. AUC scores were shown. The BRCA scores calculated based on germline *BRCA1/2* mutations achieved the best AUCs. (**c**) Boxplot for BRCA scores calculated by integrating BRCAness profile and gene expression profile offered by GSE50567 which contains profiles for familial *BRCA1* and *BRCA2* (BRCA), other familial with non-*BRCA* mutations, sporadic and normal breast cancer samples. Whitney Wilcoxon test P-values were listed. (**d**) Same with (**c**) but for GSE27830 which contains profiles for four familial breast cancer samples including *BRCA1*, *BRCA2*, *CHEK2* and other mutations (non-mutations of aforementioned genes). (**e**) Same with (**c**) but for GSE19177 which contains profiles for three groups of familial breast cancer samples including *BRCA1*, *BRCA2* and non-*BRCA1/2* mutations.

We then examined whether BRCA score could distinguish familial breast cancer patients from sporadic patients. Because the Larsen *et al.* dataset^32^ was utilized to define three BRCAness characteristic profiles, we first compared their performance using this dataset. We calculated sample-specific BRCA scores for the 33 *BRCA1*-mutant familial samples, the 22 *BRCA*2-mutant familial samples, and the 128 sporadic samples using the *BRCA1*, *BRCA*2 and the *BRCA1/2*- profiles, respectively (Fig. 2a). We found that *BRCA1*-mutant (Mann-Whitney Wilcoxon test P = 6e-13) and *BRCA2*-mutant (Mann-Whitney Wilcoxon test P = 6e-4) familial samples have significantly higher BRCA scores than sporadic samples when using all three BRCAness profiles (Fig. 2a). To further evaluate the predictive accuracy of the BRCA score, we trained a binary classification model to classify familial breast tumors from sporadic tumors (Fig. 2b). When using the *BRCA1*-based profile, the calculated BRCA scores could clearly discriminate familial *BRCA1*-mutant tumors from sporadic ones with an area under the curve (AUC) 0.901 (Fig. 2b, left). Similarly, the BRCA scores were able to distinguish familial *BRCA2*-mutant tumors from sporadic ones (AUC = 0.718), and all familial *BRCA1*/*2*-mutant tumors from sporadic ones (AUC = 0.828). Consistent observations were showed in the classifications utilizing the *BRCA2*-based (Fig. 2b, middle) and the *BRCA1/2*-based (Fig. 2b, right) BRCAness profiles. These results indicate that the calculated BRCA score can distinguish familial breast cancer patients from sporadic ones with high accuracy and can capture differences in the BRCAness phenotype. A high BRCA score implies high likelihood of carrying a *BRCA1*/*2* germline mutation and thus a more deficient HR pathway. Notably, the calculated BRCA scores using the *BRCA1*/*2*-based profile achieved the best classification accuracy when comparing familial breast tumors from sporadic ones (Fig. 2b, right, AUC = 0.861 for *BRCA1* vs. sporadic, ACU = 0.809 for *BRCA2* vs. sporadic and AUC = 0.84 for *BRCA1*/*2* vs. sporadic). Based on these results, we applied the BRCA scores calculated using the *BRCA1*/*2*-based profile for subsequent analyses.

Moreover, we utilized the GSE50567^33^ dataset to further test the ability of the BRCA score to classify breast cancer patients. This dataset contained 12 *BRCA1*- and 1 *BRCA2*-mutated hereditary breast tumors, 8 BRCAx (non-*BRCA1/2* mutations, donated as non-BRCA) hereditary breast tumors, 14 sporadic breast cancer samples and 6 normal samples. However, we found that 5 sporadic and 1 BRCAx samples had methylated *BRCA1* promoters which could result in transcriptional silencing of *BRCA1* ^34^ and a reduction in HR pathway activity. Therefore, we combined these 6 patients with the 12 *BRCA1*- and 1 *BRCA2*-mutated hereditary breast cancer patients into one group (donated as “*BRCA*”). The comparison of BRCA scores showed that patients in the *BRCA* group have higher BRCA scores than normal breast samples (Fig. 2c, Mann-Whitney Wilcoxon test P = 6e-06) and sporadic tumors (Mann-Whitney Wilcoxon test P = 0.04). Moreover, patients carrying *BRCA* mutations had higher BRCA scores than the other familial tumors with non-*BRCA* mutations (Mann-Whitney Wilcoxon test P = 7e-04). Furthermore, we observed similar results in two additional familial breast cancer datasets. The GSE27830^35–37^ dataset provided gene expression profiles for 155 familial primary breast cancer samples including 47 *BRCA1-*, 6 *BRCA2-*, 26 *CHEK2-* mutant samples and 76 samples without mutations in these three genes. Due to the limited number of *BRCA2*-mutant patients, we merged *BRCA1*- and *BRCA2*-mutant patients into one group. Based on the *BRCA1/2*-based profile, we calculated BRCA scores to patients in this dataset and found that patients with *BRCA1/2* mutations have significantly higher BRCA scores than those with *CHEK2* (Mann-Whitney Wilcoxon test P = 2e-05) and other gene (Mann-Whitney Wilcoxon test P = 6e-05) mutations (Fig. 2d). We found similar results in the GSE19177 dataset^38,39^ which contains expression profiles for 19 *BRCA1*, 30 *BRCA2* and 25 non-*BRCA1*/*2* mutation familial breast cancer samples (Fig. 2e). Again, patients with *BRCA1* mutations had higher BRCA scores than those with non-*BRCA1*/*2* mutations. These observations suggest that the calculated BRCA score is an effective classifier to distinguish familial breast tumors from sporadic tumors and to identify *BRCA1* or *BRCA2* mutated patients within familial breast cancer patients. The score is negatively correlated with the deficient HR pathway activity caused by *BRCA1* or *BRCA2* mutation.

### Association of BRCA score with genomic features

Because BRCAness significantly correlates with genomic instability^15^, we extended our computational framework to TCGA breast cancer datasets to further examine the correlation of BRCA score with genomic features. First, we investigated the association between BRCA score and somatic mutations. By comparing the difference in BRCA score between mutated and non-mutated samples for each gene, we found that somatic mutation status of three genes, *TP53* (Mann-Whitney-Wilcoxon Test P = 2e-30), *PIK3CA* (Mann-Whitney-Wilcoxon Test P = 1e-16) and *CHD1* (Mann-Whitney-Wilcoxon Test P = 2e-17), were significantly correlated with the BRCA score. Specifically, patients with higher BRCA scores were more likely to carry *TP53* mutations (Supplementary Fig. S1). In contrast, patients with lower BRCA scores were more likely to harbor mutated *PIK3CA* and *CHD1* (Supplementary Fig. S1). Then, we examined the overall association between BRCA score and CNV in breast cancer samples. According to TCGA CNV dataset, we divided genes into deletion (CNV < 1.2), normal and duplication (CNV > 2.8) groups. Ranking TCGA patients based on their BRCA scores, we found that high BRCA scores correlate with CNV (Supplementary Fig. S1). Last, by calculating the z scores across CpG sites for each patient, we found that BRCA scores are overall associated with DNA methylation (Supplementary Fig. S1).

**Figure 3:**
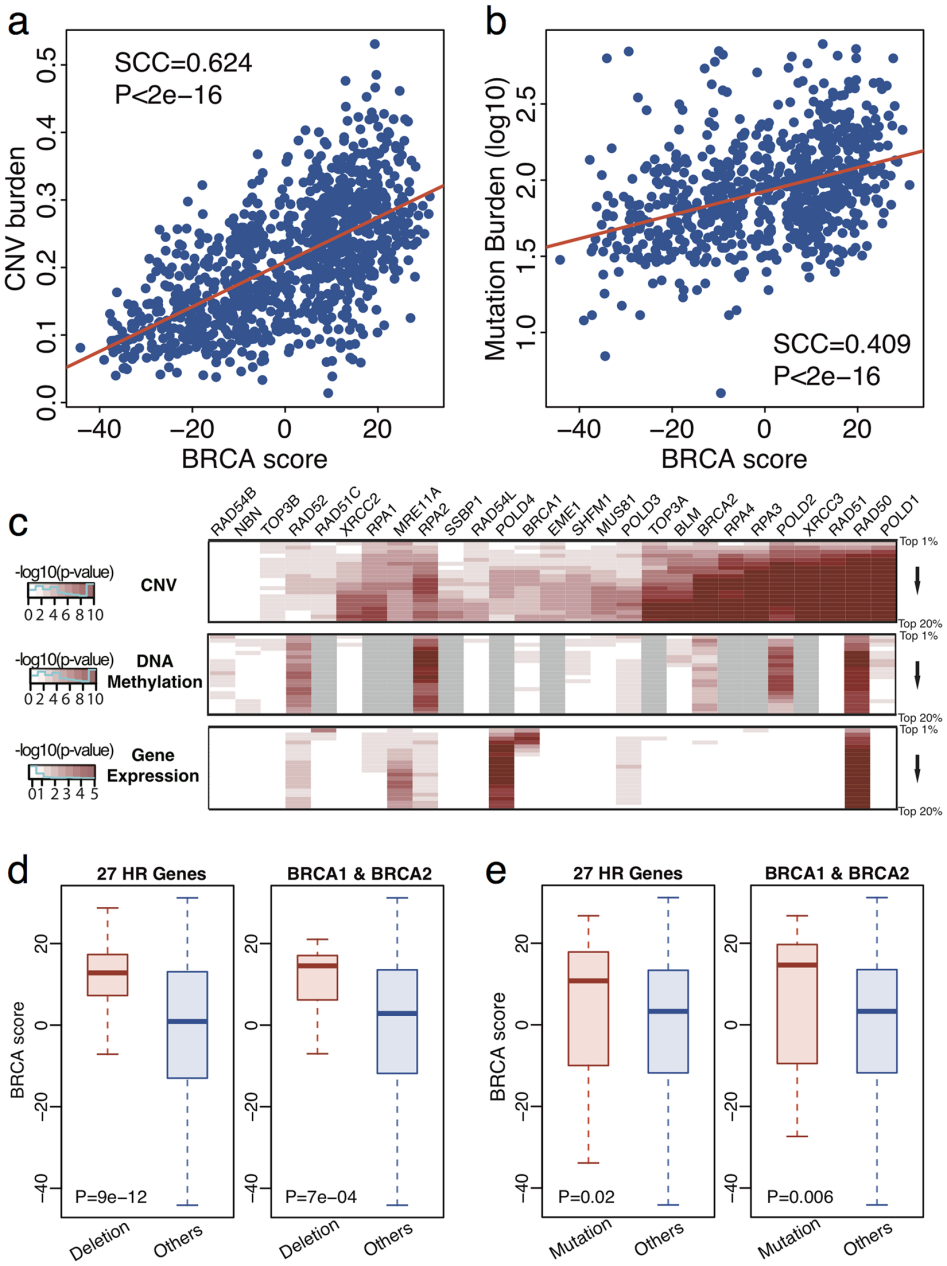
BRCA score correlates with breast cancer genomic features. (**a**) Correlation between BRCA scores and CNV burden. Spearman correlation coefficient and corresponding P-value were listed. (**b**) Correlation between BRCA scores and log-10 transferred mutation burden. Spearman correlation coefficient and corresponding P-value were listed. (**c**) We ranked TCGA breast cancer patients based on their BRCA scores in a decreasing order. We then compared the difference between top ranked (from 1% to 20%) patients and the remaining according to CNV, DNA methylation and gene expression of the 27 HR genes. The differences were calculated through the Mann-Whitney-Wilcoxon Test. Negative log-10 transferred P-values were shown. (**d**) Boxplot for BRCA scores in patients with 27 HR genes deletions and scores in the rest patients. And Boxplot for BRCA scores in patients with only *BRCA1* and *BRCA2* deletions and scores in the rest patients. Mann-Whitney-Wilcoxon Test P-values were listed. (**e**) Boxplot for BRCA scores in patients with 27 HR genes mutations and scores in the rest patients. And Boxplot for BRCA scores in patients with only *BRCA1* and *BRCA2* mutations and scores in the rest patients. Mann-Whitney-Wilcoxon Test P-values were listed.

Then, we compared the BRCA scores of TCGA breast cancer samples with their CNV burden and mutation burden, respectively. We observed that patients with a higher BRCA score had higher CNV burden (Fig. 3a, r = 0.624) and higher mutation burden (Fig. 3b, r = 0.409). These results imply that the BRCA scores are positively correlated with genomic instability. This might be because a high BRCA score indicates a defective HR DNA repair pathway which can result in a more abnormal genome. Since BRCA scores infer HR pathway activity, we next focused on 27 HR genes defined by the KEGG database^40^ to reveal the correlation between BRCA score and their genomic features. First, we ranked TCGA breast cancer patients based on their BRCA scores in a decreasing order. Second, we compared differences in CNV, DNA methylation, and gene expression of the 27 HR genes between top ranked patients (top 1% to 20%) and low ranked patients (remaining patients) (See Methods). We observed that patients with higher BRCA scores exhibited significant copy number deletion (CNV < 1.2) of the 27 HR genes compared to those with lower scores (Fig. 3c). In contrast, we observed no significant changes in DNA methylation or gene expression of the 27 HR genes between top ranking and low ranking patients. These results suggest that CNV is the primary driver of HR pathway inactivation, while DNA methylation and gene expression play minor roles in determining pathway activity. Moreover, we compared the BRCA scores for patients with 27 HR genes copy number deletion (CNV < 1.2) to the rest patients. Higher BRCA scores were observed in patients with deletion compared to the others (Fig. 3d, Mann-Whitney-Wilcoxon Test P = 9e-12). When only focusing on the two key pathway genes, *BRCA1* and *BRCA2*, we observed that copy number deleted patients have higher BRCA scores (Fig. 3d, Mann-Whitney-Wilcoxon Test P = 7e-04). However, the p-value for comparison using *BRCA1* and *BRCA2* was lower than that using the 27 HR genes which suggests that the deletion of other HR pathway genes might contribute more to effect HR pathway activity (Fig. 3d). In addition to CNV, we observed consistent results when comparing somatic mutation status with BRCA score. Namely, patients with mutations in the 27 HR genes (n = 68) exhibited higher BRCA scores compared to patients without a mutation in either of the 27 HR genes (Fig. 3e, Mann-Whitney-Wilcoxon Test P = 0.02). Moreover, patients with mutated *BRCA1* or *BRCA2* (n = 25) exhibited higher BRCA scores compared to non-mutant samples (Fig. 3e, Mann-Whitney-Wilcoxon Test P = 0.006).

### BRCA score predicts breast cancer patient prognosis

**Figure 4:**
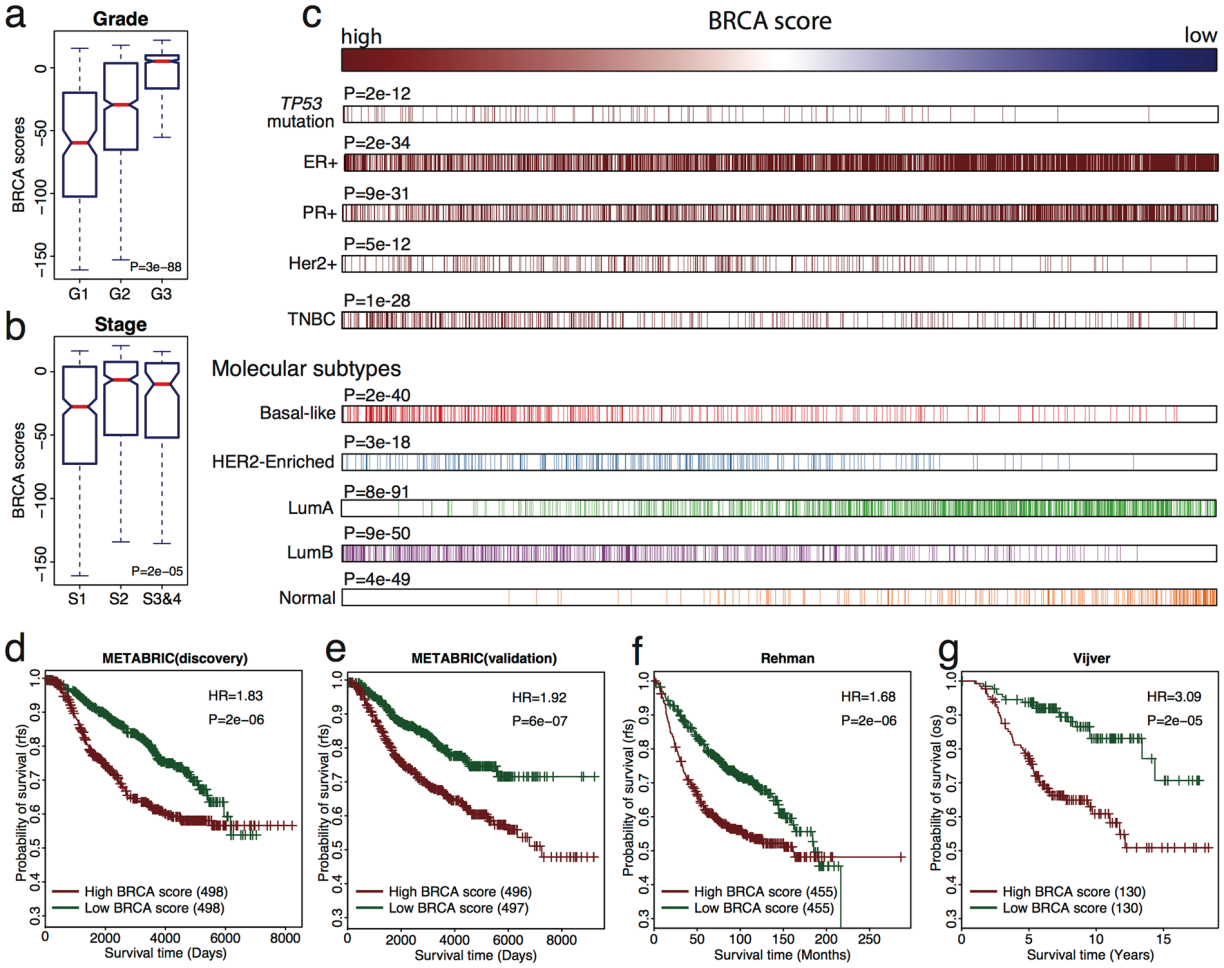
BRCA score predicts prognosis for breast cancer patients. (**a**) Boxplot for BRCA scores of patients in different grades. Mann–Whitney U-test P-value was listed. (**b**) Same as (**a**) but for different cancer stages. (**c**) Distribution of BRCA scores based on breast cancer subtypes. Then, we ranked the BRCA scores and showed the correlations with *TP53* mutation, ER status, PR status, Her2 status, triple negative breast cancer (TNBC) and molecular breast cancer subtypes. We compared BRCA scores in *TP53* mutation vs. *TP53* wild type, ER+ vs. ER−, PR+ vs. PR−, Her2+ vs. Her2−, TNBC vs. the other breast cancer samples, Basal-like vs. non-Basal-like, HER2-Enriched vs. non-HER2-Enriched, Luminal A vs. non-Luminal A, Luminal B vs. non-Luminal B and Normal vs. non-Normal patients. P-values were calculated using Mann–Whitney Wilcoxon test. (**d**–**g**) Kaplan-Meier plots for BRCA scores comparison of the (**d**) discovery, (**e**) validation datasets in the METABRIC dataset, (**f**) Ur-Rehman dataset, (**g**) Vijver dataset. Patients with low BRCA scores (green curve) had better survival than those with high BRCA scores (red curve). Hazard ratio (HR) and log-rank test P-value were shown.

We next sought to evaluate whether the BRCA score could predict survival outcomes of breast cancer patients. First, we calculated the BRCA scores using our method for patients from the METABRIC cohort, which provides the most comprehensive breast cancer gene expression dataset that is accompanied by exhaustive clinical records for 1,992 primary breast cancer patients^41^. We next compared the association of BRCA scores with clinic pathological characteristics including grade and stage which are well-established clinical indicators of disease severity. Our results show that the calculated BRCA score was positively correlated with tumor grade, with higher grade tumors showing higher BRCA scores (Mann–Whitney U-test, P = 3e-88; Fig. 4a). Likewise, we observed that patients with stage 2–4 tumors exhibited significantly higher BRCA scores compared to patients with stage 1 tumors (Mann–Whitney U-test, P = 2e-05; Fig. 4b). These results indicate that BRCA score reflects the aggressiveness of tumors and can serve as an indicator of disease progression.

Moreover, we ranked the BRCA scores of patients from high to low and analyzed the distribution of patients based on several variables including *TP53* mutation, ER status, progesterone receptor (PR) status, HER2 status, triple negative status, and molecular subtype (Fig. 4c and Supplementary Fig. S2). Patients carrying *TP53* mutations were tended to have higher BRCA scores compared to those with wild-type *TP53* (Mann–Whitney Wilcoxon test P = 2e-12) which is consistent with our result in the TCGA dataset. Moreover, we found that ER+ patients have a higher likelihood of lower BRCA scores compared to those with ER− (Mann–Whitney Wilcoxon test P = 2e-34). Similar, PR+ patients had lower calculated BRCA scores than those with PR− (Mann–Whitney Wilcoxon test P = 9e-31). In contrast, patients with HER2+ tended to have higher BRCA scores (Mann–Whitney Wilcoxon test P = 5e-12). Moreover, triple negative breast cancer (TNBC) was more likely to be present in patients with high BRCA scores (Mann–Whitney Wilcoxon test P = 1e-28). Furthermore, we compared the BRCA scores in patients with Basal, HER2-enriched, Luminal A, Luminal B, or Normal-like tumors. Patients with higher BRCA scores had higher risk to be predicted as Basal-like (Mann–Whitney Wilcoxon test P = 2e-40), HER2-enriched (Mann–Whitney Wilcoxon test P = 3e-18) and Luminal B (Mann–Whitney Wilcoxon test P = 9e-50) breast cancers. However, patients with lower BRCA scores were more likely to be Luminal A (Mann–Whitney Wilcoxon test P = 8e-91) and Normal-like (Mann–Whitney Wilcoxon test P = 4e-49) breast cancer. Additionally, normal-like breast cancer patients tend to have the lowest BRCA scores.

After demonstrating that BRCA scores vary across samples with different clinical features, we evaluated whether BRCA scores could predict breast cancer prognosis. Using BRCA score as the independent variable, we performed survival analyses in the METABRIC discovery and validation datasets. Namely we divided patients into high and low BRCAness categories by stratifying on median BRCA score. We observed that high BRCAness was associated with a significant increase in mortality risk in the discovery cohort (Fig. 4d, P = 2e-06) with a hazard ratio (HR) of 1.83. Similarly, this result was reproduced in the METABRIC validation dataset (Fig. 4e, P = 6e-07, HR = 1.92). In addition, we found that the BRCA score was predictive of prognosis in ER+ patients in both the discovery (HR = 1.63, P = 0.001) and validation (HR = 2.24, P = 1e-06) datasets, with BRCA score being associated with increased mortality risk (Supplementary Fig. S3). In ER− patients, exhibited lower prognostic ability potentially due to high heterogeneity of ER− tumors^42^. Interestingly, we found that TNBC patients with high BRCA scores exhibited improved survival in the discovery dataset (Supplementary Fig. S4, HR = 2.21, P = 0.008).

To further evaluate the reproducibility of the BRCA score to predict survival, we applied the weight profiles to interrogate and perform survival analysis in two additional breast cancer datasets by Ur-Rehman *et al*.^43^ and Vijver *et al*.^44^. Our results remained consistent in that patients with high BRCA scores had worst prognosis than those with low BRCA scores (Ur-Rehman, Fig. 4f, P = 2e-06, HR = 1.68; Vijver, Fig. 4g, P = 2e-05, HR = 3.09). Additionally, the BRCA score was able to better predict mortality in patients with ER+ tumors compared to patients with ER− tumors in both datasets (Supplementary Fig. S4). Furthermore, to determine if the BRCA score could provide additional prognostic information to traditional clinicopathological variables, we fitted a multivariate Cox regression model to the METABRIC dataset using BRCA scores and clinical variables including age, ER status, Her2 status, stage, and grade as independent variables. Our results show that BRCA scores (HR = 1.63, P = 3e-03) remain predictive to prognosis even after considering other clinical information (Supplementary Table S2).

### BRCA score predicts patient response to neoadjuvant chemotherapy

**Figure 5:**
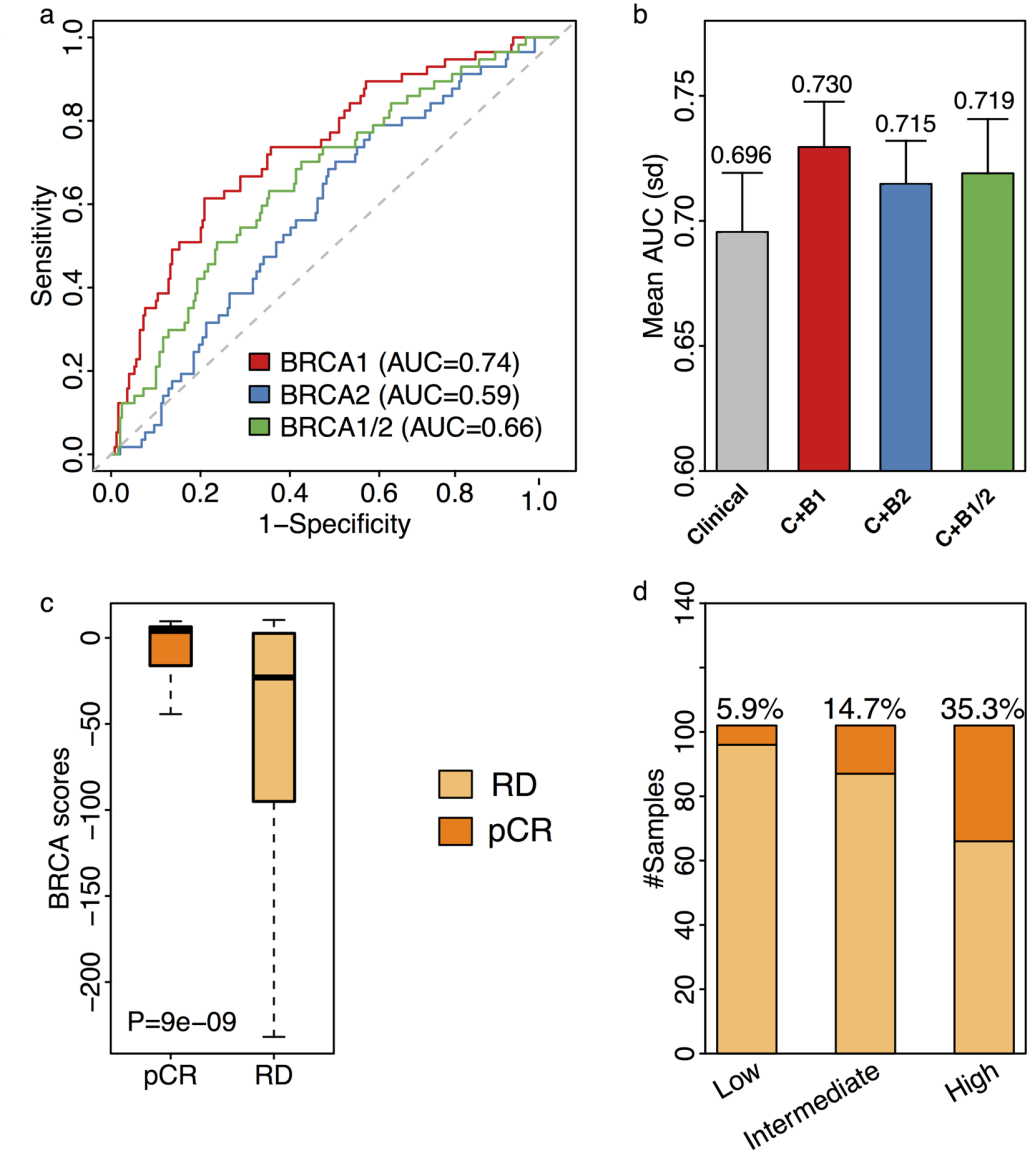
BRCA score predicts chemotherapy for breast cancer samples. (**a**) ROC curves for the accuracy of classifying pathologic complete response (pCR) from residual disease (RD) breast cancer patients. The BRCA scores were calculated using gene expression of germline mutate-*BRCA1* (red), germline mutate-*BRCA2* (blue), germline mutate-*BRCA1*/*2* (green) as reference to compare with those of sporadic ones, respectively. AUC scores were listed. (**b**) Barplot for the mean AUC scores of 10-fold cross validation using clinical information (gray), clinical information + *BRCA1* based BRCA scores (C+*B1*, red), clinical information + *BRCA2* based BRCA scores (C+*B2*, blue) and clinical information + *BRCA1/2* based BRCA scores (C+*B1/2*, green) to classify pCR from RD patients. The corresponding average AUCs were listed above each bar. Standard deviations were plotted with the error bars. (**c**) Boxplot for BRCA scores in patients with pCR (dark orange) and RD (chardonnay). BRCA scores were calculated using the *BRCA1*-based profiles. Mann-Whitney-Wilcoxon Test P-value was listed. (**d**) Barplot for pCR patients’ fractions in different BRCA score groups. The chardonnay bar is RD patients and the dark orange bar is pCR patients. Corresponding pCR rate of each group were listed above the bars.

Since BRCAness is a biomarker for responsiveness of chemotherapy^15^, we further examined whether the BRCA score could predict patient response to neoadjuvant chemotherapy. We calculated BRCA scores for patients in the Hatzis breast cancer dataset, which includes treatment response information to neoadjuvant taxane-anthracycline chemotherapy for 508 breast cancer samples^45^. BRCA scores were calculated with the *BRCA1*-, *BRCA2*- and *BRCA1*/*2*-based profiles, respectively. First, we used BRCA scores to classify patients achieving pathologic complete response (pCR) and those with residual disease (RD). The results showed that using the *BRCA1*-based profile to calculate BRCA scores (AUC = 0.74) achieves the best accuracy for classification compared to using the *BRCA2*- (AUC = 0.59) or *BRCA1*/*2*-based (0.66) profiles (Fig. 5a). Moreover, to demonstrate that the BRCA score contributes to clinicopathological variables in predicting treatment response, we constructed random forest models to classify pCR patients from patients with RD (Fig. 5b). For one model, we only used the clinical variables including age, ER status, PR status, HER2 status, grade, stage, node information as predictors. We compared this model to a second model where we include both BRCA scores and clinicopathological variables as predictors. We observed that BRCA scores calculated from the *BRCA1*-based profile yielded the highest average AUC (AUC = 0.73) compared to scores generated from there *BRCA2*- (AUC = 0.715) and *BRCA1*/*2*-based (AUC = 0.719) profiles. These results suggest that using the BRCA scores calculated using *BRCA1*-based BRCAness profiles achieve the best accuracy for classification which in line with the previously studies^46,47^. Therefore, we applied those *BRCA1*-based BRCA scores to the rest chemotherapy prediction.

Moreover, applying the BRCA scores calculated with *BRCA1*-based BRCAness profile, we found that pCR patients have higher BRCA scores than RD patients (Fig. 5c, Mann-Whitney-Wilcoxon Test P = 3e-06). In addition, we observed consistent results in different breast cancer subtypes. For patients with ER+ (Mann-Whitney-Wilcoxon Test P = 0.002), ER− (Mann-Whitney-Wilcoxon Test P = 0.002) and TNBC (Mann-Whitney-Wilcoxon Test P = 0.02), we entirely found that pCR patients have higher BRCA scores compared to the RD patients (Supplementary Fig. S5). Moreover, we divided patients into low, intermediate, and high BRCA score groups and tested the fraction of pCR patients in each group. As shown in Fig. 5d, there were 5.9%, 14.7% and 35.3% pCR patients in these 3 groups. Moreover, patients with the high BRCA scores were 6-fold more likely to be pCR compared to those with the low scores. These observations suggest that our BRCA score could be utilized as a biomarker which can predict the response to neoadjuvant chemotherapy. Therefore, BRCA score could apply to practical clinical application helping doctors to improve treatment decisions and prognosis determinations.

### Apply the BRCAness characteristic profile to ovarian cancer

**Figure 6:**
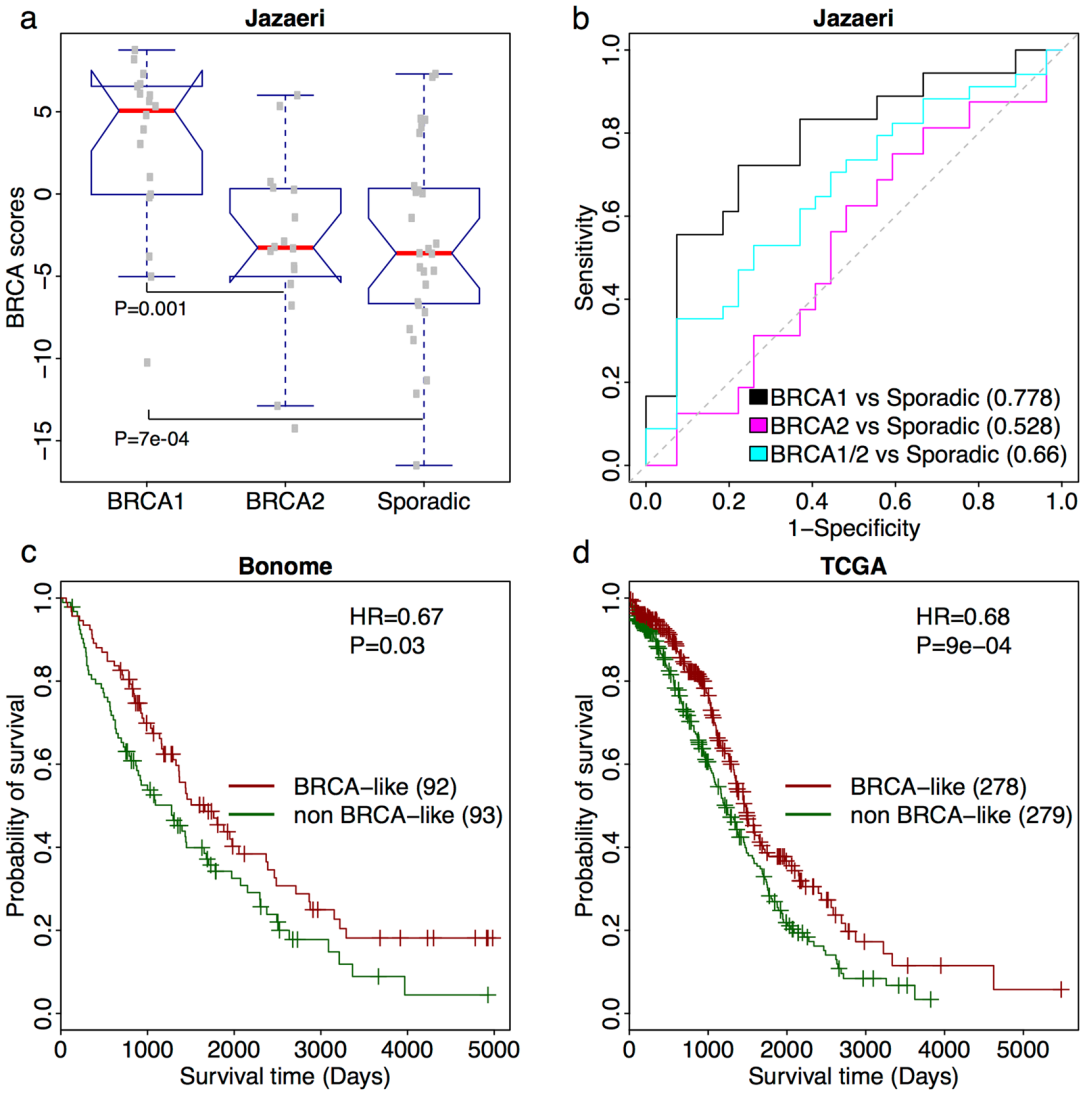
BRCA scores implements in ovarian cancer. (**a**) Boxplot for BRCA scores in germline mutate-*BRCA1*, germline mutate-*BRCA2* and sporadic ovarian cancer samples. Patients carrying germline *BRCA1* mutations had much higher BRCA scores than sporadic ones (Mann-Whitney-Wilcoxon Test P = 7e-04). (**b**) ROC curves for the accuracy of classifying familial from sporadic ovarian cancer patients using BRCA scores. Black curve: comparison of patients with germline *BRCA1* mutations to sporadic ones. Magenta curve: comparison of patients with germline *BRCA2* mutations to sporadic ones. Cyan curve: comparison of patients with germline *BRCA1* and *BRCA2* mutations to sporadic ones. AUC scores were shown. (**c**) Kaplan-Meier plots for patients’ prognosis in the Bonome ovarian cancer dataset. BRCA-like patients (red curve) had better survival than non BRCA-like ones (green curve). Hazard ratio (HR) and log-rank test P-value were shown. (**d**) Same as (**c**) but for TCGA ovarian cancer samples.

Previous studies have shown that BRCAness also plays an important role in ovarian cancer^48,49^. Briefly, due to the sensitivity of chemotherapy^50^, BRCAness is significantly associated with ovarian cancer outcome^51^. Thus, we evaluated whether the BRCAness characteristic profile generated using breast cancer profiles can be applied to interrogate pathway activity in ovarian cancer. Using gene expression profiles from ovarian cancer tumor samples provided by Jazaeri *et al*.^52^, we first calculated sample-specific BRCA scores for each patient in the dataset using the *BRCA1*/*2*-based breast cancer profile. The Jazaeri dataset^52^ contained 18 germline *BRCA1* mutated, 16 germline *BRCA*2 mutated and 27 sporadic ovarian cancer samples. By comparing the BRCA scores, we found that patients with *BRCA1* germline mutations have higher BRCA scores than either patients with *BRCA*2 germline mutations (Fig. 6a, Mann-Whitney-Wilcoxon Test P = 0.001) or sporadic ones (Mann-Whitney-Wilcoxon Test P = 7e-04) which are consistent with their results^52^. Moreover, using the BRCA score could classify BRCA1-mutated familial ovarian cancer tumors from sporadic ones with high accuracy (AUC = 0.778). When classifying *BRCA1* or *BRCA*2 mutant samples from sporadic cancers we achieved an AUC = 0.660; however, classification of only germline *BRCA*2 mutant tumors was inaccurate (AUC = 0.528) (Fig. 6b). These observations suggest that the BRCA score using the BRCA1 profile can be applied as a prognostic biomarker in ovarian cancer.

Therefore, we tested whether the BRCA score could predict ovarian cancer prognosis using the Bonome *et al*.^53^, TCGA^54^ and the Yoshihara *et al*.^55^ datasets, which contain 185, 557, and 260 patients, respectively. After calculating a BRCA score for each patient, we divided them into BRCA-like and non BRCA-like groups using median BRCA score as the cutoff. Interestingly, we observed that BRCA-like patients exhibited decreased mortality risk in both the Bomone (Fig. 6c, HR = 0.67, P = 0.03) and TCGA datasets (Fig. 6d, HR = 0.68, P = 9e-04). A similar trend was observed using the Yoshihara dataset (Supplementary Fig. S6, HR = 0.9) but the difference between two groups was not significant (P > 0.05). These results were in line with the previous study^28,56^, which suggest the BRCAness profile calculated with breast cancer expression profiles can be applied to ovarian cancer.

## Discussion

Our analyses indicate that BRCAness can be caused by different genomic mechanisms including somatic mutations, aberrant methylation, deletions and downregulated expression of genes involved in DNA repair. It is notable that gene deletions might contribute more to BRCAness than other genomic changes in sporadic breast cancers (Fig. 3a). In line with this observation, we found that the BRCA score of samples is more correlated with CNV burden than with mutation burden (Fig. 3b,c). However, this observation may not justify the use of CNV-based supervised classification models to assess BRCAness^27^. Although BRCAness is associated with a high level of genome instability as manifested by high CNVs, genomic deletions or amplifications that occur may not exist in most BRCA-like samples to provide informative predictors for these classification models due to the heterogeneity of sporadic tumor samples–BRCAness can be caused by inactivating different genes via distinct mechanisms, such as DNA methylation and mutations. To address this shortcoming, we developed a framework that measures BRCAness based on transcriptomic profiles which capture the final downstream readout of genomic lesions that abrogate HR pathway activity. As such, a key question is why can the BRCAness profile be defined by comparing *BRCA1*/2-mutant familial tumor samples with sporadic samples? The familial breast tumor samples used for defining the BRCAness profile inherit a copy of defective *BRCA1*/2 gene, predispose them to breast cancer when the function of the other allele is lost (again, this can be caused by different genomic mechanisms). Thus, the familial breast tumor samples carrying *BRCA1*/2 germline mutations represent a ‘homogenous’ set of cancers that are driven by HR pathway inactivation. As a result, it is possible to create a characteristic BRCAness gene expression profile that encodes the transcriptomic changes accoiated with known loss of HR pathway activity. Breast tumor’s HR pathway activity could be inferred via this profile where a high BRCA score that indicates low activity. Although the familial breast tumor samples provide an ideal positive dataset for defining BRCAness profile, the sporadic samples may not be a good negative dataset since some of these sporadic samples may be BRCA-like causing by other mechanisms such as BRCA1 promoter DNA methylation, deletions of other HR genes or post-transcriptional regulation. Thus, we would expect to further improve our analysis by excluding these BRCA-like samples to achieve a more accurate BRCAness profile.

In the TCGA breast cancer data, 13 and 14 samples contain at least one somatic mutation in *BRCA1* and *BRCA*2, respectively. We mapped these mutations to the protein sequence of BRCA1 and BRCA2, and calculated the corresponding BRCA scores for these samples (Supplementary Fig. S7). The majority of these patients with BRCA1/2 somatic mutations are associated with positive BRCA scores, indicating lower HR pathway activities. However, nine out of 27 samples are associated with negative scores, suggesting that different mutation types in *BRCA1* and *BRCA*2 genes vary in their functional impacts. To inactivate the HR pathway, both *BRCA1*/*2* alleles have to be defective, which might occur through different mechanisms. Furthermore, it is often difficult to determine the effect of a particular mutation occurred in *BRCA1*/*2* genes. Potentially, some mutated BRCA1/2 proteins may preserve the ability to bind but cannot repair DNA, and furthermore, prevent the wild-type *BRCA1*/*2* (encoded by the other allele) from carrying out repairing functions, and therefore result in a dominant effect^57,58^ posing another layer of complexity. Therefore, the BRCA scores calculated by our framework provide a useful measurement for BRCAness in sporadic breast cancer samples.

Our analyses indicate that the BRCAness profile defined based on familial breast cancer samples can be applied to classify *BRCA1*/*2*-mutant familial versus sporadic ovarian cancer. It can also be used to assess BRCAness in sporadic ovarian tumor samples that show significant correlation with prognosis of patients. This indicates shared gene expression patterns between breast and ovarian cancer with BRCAness phenotypes. Interestingly, BRCAness is associated with poor prognosis in breast cancer but good prognosis in ovarian cancer. This might be due to the difference of treatments for these two cancer types. For ovarian cancers, it has higher likelihood to be high-grade serous carcinoma (HGSC) when the cancer was investigated^59^. It has been confirmed that HGSC is sensitive to platinum-based chemotherapy^60^. This is consistent with our conclusion that a patient with high BRCA score (high BRCA score is similar to HGSC) is more responsive to chemotherapy (Fig. 5c,d). In contrast, breast cancer is mostly driven by ER^61^ which is generally treated by hormone therapy.

## Methods

### Datasets

We collected gene expression and clinical information for breast cancer and ovarian cancer patients from 13 datasets (Supplementary Table S3). Larsen *et al*.^32^ provided the gene expression for 55 familial and 128 sporadic breast tumor samples. In the familial samples, 33 and 22 carry *BRCA1* and *BRCA2* germline mutations. We downloaded this data from the Gene Expression Omnibus (GEO) database with accession number GSE40115. The GSE50567^33^ contained 12 *BRCA1*- and 1 *BRCA2*-mutated hereditary breast tumors, 8 BRCAx (non-*BRCA1/2* mutations) hereditary breast tumors, 14 sporadic breast cancer samples and 6 normal samples. The GSE27830^35–37^ dataset provided gene expression profiles for 155 familial primary breast cancer samples including 47 *BRCA1-*, 6 *BRCA2-*, 26 *CHEK2-* mutant samples and 76 samples without mutations in these three genes. The GSE19177 dataset^38,39^ contained expression profiles for 19 *BRCA1*, 30 *BRCA2* and 25 non-*BRCA1*/*2* mutation familial breast cancer samples. The METABRIC breast cancer dataset^41^ contained gene expression and exhaustive clinical profiles for 1,992 tumors which was downloaded from the European Genome Phenome Archive with accession number EGAS00000000083. The METABRIC dataset provided *TP53* status for 820 patients including 99 patients with *TP53* mutations and 721 patients with wild type *TP53*. The Ur-Rehman dataset^43^ contained 1,170 samples integrated from 5 existed breast cancer datasets^62–66^ and was downloaded with accession number GSE47561. The Vijver dataset^44^ was downloaded from the Netherlands Cancer Institute (http://ccb.nki.nl/data/) and contained gene expression and clinical profiles for 295 breast cancer patients. The Hatzis dataset^45^ contained the response to neoadjuvant chemotherapy for 508 HER2-negative breast cancer samples with an accession ID GSE25066. Additionally, we applied our analyses to three OV datasets. Jazaeri dataset^52^ contained 18 germline mutated *BRCA1*, 16 germline mutated *BRCA2* and 27 sporadic ovarian cancer samples accessing with GSE82007. Bonome *et al*.^53^ provided the gene expression profile for 185 late-stage and high-grade ovarian cancer patients with the accession number GSE26712. Yoshihara *et al*.^55^ contained 260 Japanese advanced-stage ovarian cancer patients which was downloaded with an accession number GSE32062. Besides, additional TCGA datasets for breast cancer^67^ and ovarian cancer^54^ samples were collected from FIREHOSE Broad institute (https://gdac.broadinstitute.org/).

### Define characteristic profiles for homologous recombination DNA repair pathway

The gene expression dataset generated by Larsen *et al*.^32^ was used to define the characteristic profile for BRCAness in breast cancer. This data was provided as log transformed expression at probeset level. We converted the data into gene expression data based on the probeset annotation. For genes with multiple probesets, the probeset with the highest average hybridization signals across all samples was selected to represent a gene.

Following that a logistic linear regression model was constructed for each gene to evaluate its power for differentiating *BRCA1*/*2*-mutant samples from sporadic samples while adjusting several important clinical variables. Specifically, the following model is used:

$$\begin{array}{ccc}{\rm{l}}{\rm{n}}({p}_{j}/(1-{p}_{j})) & = & \alpha +\beta \ast {{\rm{g}}{\rm{e}}{\rm{n}}{\rm{e}}}_{i}+{\gamma }_{1}\ast {\rm{a}}{\rm{g}}{\rm{e}}+{\gamma }_{2}\ast {\rm{g}}{\rm{r}}{\rm{a}}{\rm{d}}{\rm{e}}+{\gamma }_{3}\ast {\rm{t}}{\rm{u}}{\rm{m}}{\rm{o}}{\rm{r}}\,{\rm{s}}{\rm{i}}{\rm{z}}{\rm{e}}\\  &  & +{\gamma }_{4}\ast {\rm{E}}{\rm{R}}\,{\rm{s}}{\rm{t}}{\rm{a}}{\rm{t}}{\rm{u}}{\rm{s}}+{\gamma }_{5}\ast {\rm{H}}{\rm{E}}{\rm{R}}2\,{\rm{s}}{\rm{t}}{\rm{a}}{\rm{t}}{\rm{u}}{\rm{s}},\end{array}$$

where *p*_*j*_ is the probability of the sample *j* is *BRCA1*/*2*-mutant.

For each sample, the model calculated a coefficient and its p-value for each variable. The sign of *β* indicates whether *gene*_*i*_ has higher expression levels in familial (if *β* > 0) or sporadic (if *β* <= 0) samples. The p-value for *β* indicates the capability of the gene to discriminate familial from sporadic samples, the smaller p-value the more significant discriminative power. Logistic linear regression was performed for all genes and the resulting beta coefficients were collected into a vector *B*_*j*_ = {*β*_1_, *β*_2_,…, *β*_n_} and the p-values were collected into a vector *P*_*j*_ = {*p*_1_, *p*_2_, …, *p*_n_}, where n is the total number of genes. Based on the two vectors, a pair of weight profiles was generated to quantify the discriminative power of all genes to differentiate familial from sporadic samples using the following functions:

$${W}_{j}^{+}=-\mathrm{log}\,10({p}_{{\rm{i}}})\ast {\bf{I}}({\beta }_{{\rm{i}}} > 0)\,{\rm{and}}\,{{W}_{j}}^{-}=-\mathrm{log}\,10({p}_{{\rm{i}}})\ast {\bf{I}}({\beta }_{{\rm{i}}} < =0).$$

This pair of weight profiles characterize up- (*W*_*j*_^+^) and down-regulated (*W*_*j*_^−^) genes in *j*-th familial sample, respectively. **I** is the indicator function which outputs 1 if *β* >= 0 and 0 when *β* <= 0. Weights greater than 10 in these profiles were trimmed to avoid extreme values. By integrating the weight profiles cross samples, we generated *W* ^+^ and *W* ^*−*^ weight profiles, which, together, defines the characteristic profile for BRCAness. Based on the Larsen *et al*.^32^ dataset, we define three BRCAness profiles for breast cancer by comparing *BRCA1*, *BRCA*2 and *BRCA1*/2 familial samples with sporadic samples, respectively.

### Calculate sample-specific activity score for homologous recombination DNA repair pathway

The BRCAness profile weights genes in a sorted tumor gene expression profile to discriminate HR-defective samples (familial) from HR-proficient samples (sporadic). Given this profile, we apply a rank-based method to infer HR pathway activity in tumor samples based on their expression profiles. Typically, transcriptomic data for tumor samples are provided as either relative expression or absolute values dependent the platforms used for generating the data. When two channel microarray platforms are used, the resulting data represent relative expression of genes with respect to a reference sample. In contrast, one channel microarray and RNA-seq platforms generate absolute expression level of genes. In this case, we convert data into relative expression via median normalization.

Providing the relative gene expression profile for a tumor sample, we rank genes based on their expression and summarize the baseline expression of genes by referring to their weights in the BRCAness profile, resulting in a BRCA score which is a quantitative measure of HR pathway activity. The underlying rationale is that in BRCA-like samples highly expressed genes (*i*.*e*., those with greater positive or negative relative expression values) tend to have higher weights in the BRCAness profile (*W* ^+^ and *W* ^−^), while the opposite is true for the non-BRCA-like samples. This form of correlation is nonlinear and is sensitive to genes distributed at the two ends of the sorted tumor gene expression profile. A rank-based statistical algorithm called BASE^31^, which is designed specifically to measure this correlation pattern, is applied to calculate BRCA scores in tumor samples. Briefly, genes are sorted into a ranked gene list based on their relative expression, and the biased distribution of BRCAness-upregulated (with high values in *W* ^+^) and downregulated (with high values in *W* ^−^) genes in this list were examined to obtain two scores, *BS* ^+^ and *BS* ^−^, respectively. The BRCA score is defined as their difference, *BS* ^+^ − *BS* ^−^. A higher BRCA score indicates high likelihood of BRCAness and thereby lower HR pathway activity. Conversely, a lower BRCA score indicates less likelihood of BRCAness and thereby higher HR pathway activity. A similar statistical framework has been applied to integrate cancer gene expression data with gene knockdown profiles with detailed description available from Wang *et al*.^30^. The calculated BRCA scores were highly consistent with each other using *BRCA1*-, *BRCA*2- and *BRCA1*/*2*-based BRCAness profiles in all datasets we applied in our analyses (Supplementary Fig. S8).

### Pathway enrichment analysis

According to the weights in the BRCAness profiles generated by comparing familial versus sporadic breast cancer samples, we selected 300 genes with the highest positive weights as the genes up-regulated in familial tumors. Similarly, three hundred down-regulated genes in familial tumors were selected with the highest negative weights. Pathway enrichment analysis was performed based on the REACTOME database^68^. Hypergeometric test was used to calculate the p-value. Adjusted p-value was calculated by Benjamini and Hochberg method. All analyses were performed in R.

### Associate BRCA scores with patient prognosis in breast and ovarian cancer

We applied Kaplan-Meier method to compare the survival times of patients in different groups. A log-rank test P-value was calculated to estimate the difference. Median of BRCA scores was applied as a threshold to divide patients into low and high BRCA score groups. The Cox proportional regression model was used to evaluate the individual contribution of BRCA scores to predicting survival in addition to the other clinical variables including age, ER status, Her2 status, tumor stage and tumor grade. Survival analysis was performed with the R package “survival”.

### Correlate BRCA scores with genomic features

Using the gene expression profiles of TCGA breast cancer samples, we calculated a BRCA score for each sample. Because of the calculated BRCA score infers HR pathway activity, we focused on the genomic features of 27 HR genes that explains the majority of the correlation between BRCA score and genomic features including CNV, DNA methylation, and gene expression. First, we ranked TCGA breast cancer patients based on their BRCA scores in decreasing order. For CNV and gene expression, we compared the difference between that in top ranked (from 1% to 20%) patients and the remaining ones for each 27 HR genes. For DNA methylation profile, we first used the average beta value of CpGs in the promoter (from −2k to 2k of transcription start site) of a gene to represent this gene. Then, we compared the difference between that in top ranked (from 1% to 20%) patients and the remaining ones for each 27 HR genes. The Mann-Whitney-Wilcoxon Test was applied to calculate the difference. The calculations of CVN burden and mutation burden were same with our previously study^30^.

### Associate BRCA scores with patient responsiveness to neoadjuvant chemotherapy

We used random forest^69^ models to predict pCR and RD patients in the Hatzis dataset. We only considered clinical variables such as age, ER status, PR status, HER2 status, tumor grade, tumor stage, node information as predictors in the one model. In the other models, we combined the BRCA scores calculated using *BRCA1*-, *BRCA2*- and *BRCA1*/*2*-based profiles with those clinical predictors. The accuracy of prediction was investigated by calculating the AUC scores with 10-fold cross validation. The average and standard deviation of AUC scores were calculated to show the accuracy. The R package “randomForest” was used to perform these analyses. Moreover, R function “quantile” was utilized to divide patients into three groups.

## Electronic supplementary material

Supplementary files
