# Supplementary material for: Computational Investigation of Homologous Recombination DNA Repair Deficiency in Sporadic Breast Cancer
Source: Sci Rep. 2017 Nov 16;7:15742. doi: 10.1038/s41598-017-16138-2 (PMC5691048; doi:10.1038/s41598-017-16138-2)
Supplement: Supplementary file 1 — Supplementary files [file 41598_2017_16138_MOESM1_ESM.pdf]

Computational Investigation of Homologous Recombination DNA Repair Deficiency in Sporadic Breast Cancer

Yue Wang[1,2], Matthew H. Ung[2], Sharon Cantor[3] and Chao Cheng[2,4,5,*]

[1]School of Electronic Information and Communications at Huazhong University of Science and Technology, Wuhan, Hubei, 430074, China.
[2]Department of Molecular and Systems Biology, Geisel School of Medicine at Dartmouth College, Hanover, NH 03755, USA.
[3]Department of Molecular, Cell and Cancer Biology, University of Massachusetts Medical School, MA 01605, USA.
[4]Norris Cotton Cancer Center, Geisel School of Medicine at Dartmouth College, Lebanon, NH 03766, USA.
[5]Department of Biomedical Data Sciences, Geisel School of Medicine at Dartmouth College, Lebanon, NH 03766, USA.

Contents

Figure S1. BRCA scores correlate with genomic features.
Figure S2. BRCA scores correlate with different breast cancer phenotypes.
Figure S3. BRCA scores correlate with patients' prognosis.
Figure S4. BRCA scores correlate with patients' prognosis in additional datasets.
Figure S5. BRCA scores correlate with neoadjuvant chemotherapy.
Figure S6. Kaplan-Meier plots for the Yoshihara dataset.
Figure S7. BRCA scores correlate with BRCA1 and BRCA2 protein sequence.
Figure S8. BRCA scores are consistent using different BRCAness profiles.
Table S1. Pathways enriched in different gene groups.
Table S2. The result for multivariate Cox regression model.
Table S3. Summarization of datasets used in our study.

Supplementary Figure S1. BRCA scores correlate with genomic features. We ranked BRCA scores of TCGA breast cancer patients from high to low. By comparing the difference of BRCA scores of genes in different status (mutation vs. wild-type), we found three genes, *TP53* (P=2e-30), *PIK3CA* (P=1e-16) and *CHD1* (P=2e-17), are significant correlated with the calculated BRCA scores. Patients with higher BRCA scores were more likely to carry *TP53* mutations while *PIK3CA* and *CHD1* wild-type. Moreover, we found that the calculated BRCA score were associated with overall copy number variation (CNV) and DNA methylation. Specifically, patients with high BRCA scores were more likely to have CNV.

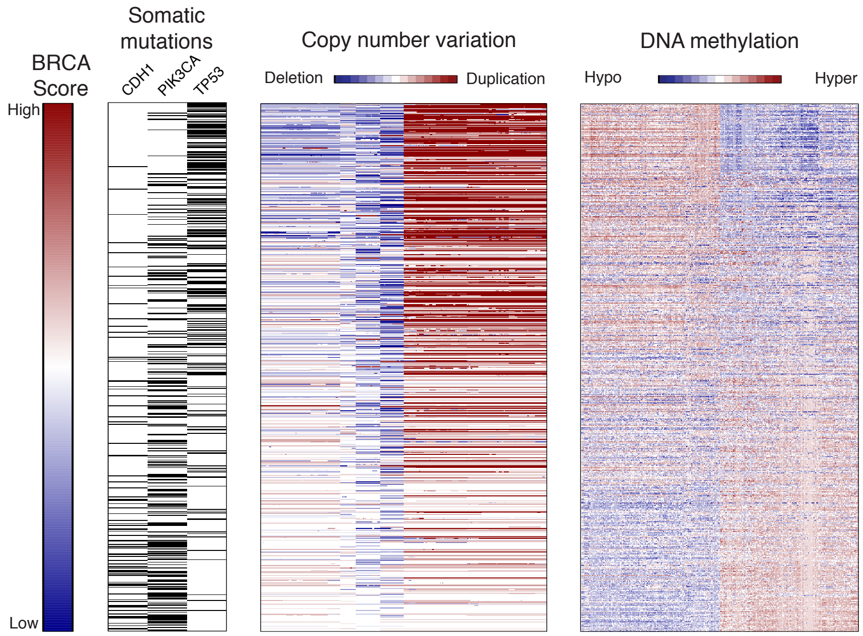

Supplementary Figure S2. BRCA scores correlate with different breast cancer phenotypes. We correlated our BRCA scores with different breast cancer phenotypes in the METABRIC dataset. Specifically, we compared BRCA scores in A) *P53* mutation vs. wild-type, B) ER+ vs. ER-, C) PR+ vs. PR-, D) HER2+ vs. HER2-, E) triple negative breast cancer (TNBC) vs. non-TNBC and F) molecular subtypes. Mann–Whitney Wilcoxon test P-values were listed. For F), the p-value perpendicular to each box means that this subtype vs. the rest patients, *e.g.* the p-value (P=2e-40) under the Basal-like box means the difference of BRCA scores between Basal-like patients vs. non-Basal-like patients.

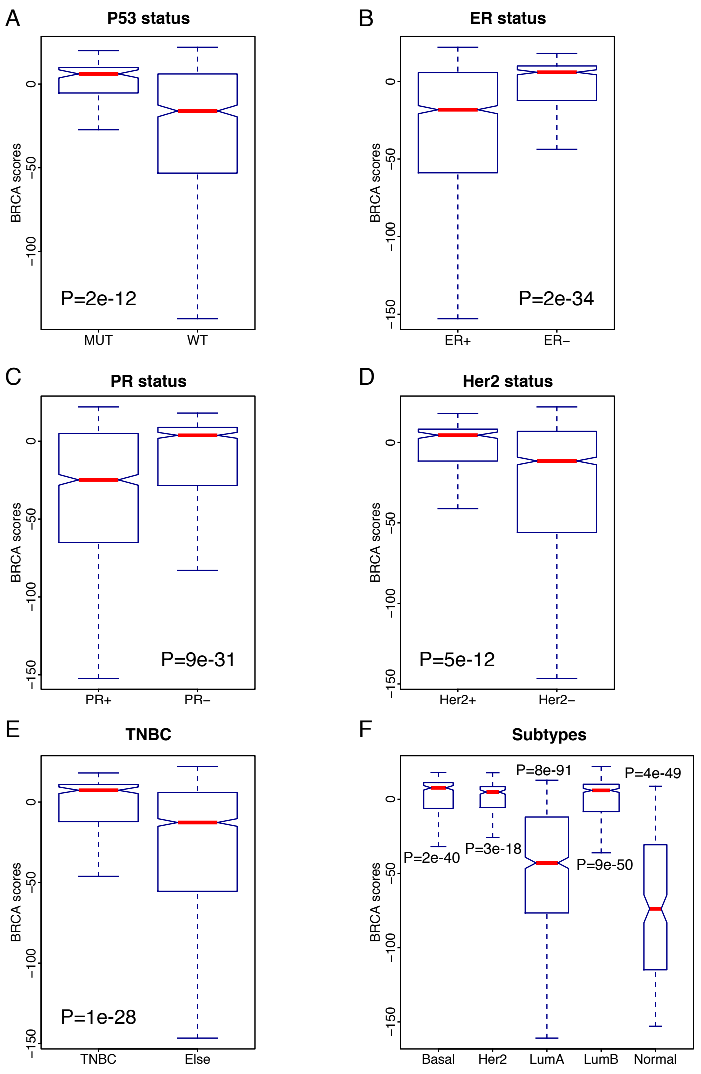

Supplementary Figure S3. BRCA scores correlate with patients' prognosis. Kaplan-Meier plots for the METABRIC discovery and validation datasets. Patients were divided into low and high BRCA score groups. Median of BRCA scores was applied as the cutoff. Red curves were patients with high BRCA scores and green curves were patients with low BRCA scores. Hazard ratio (HR) and log-rank test P-values were listed.

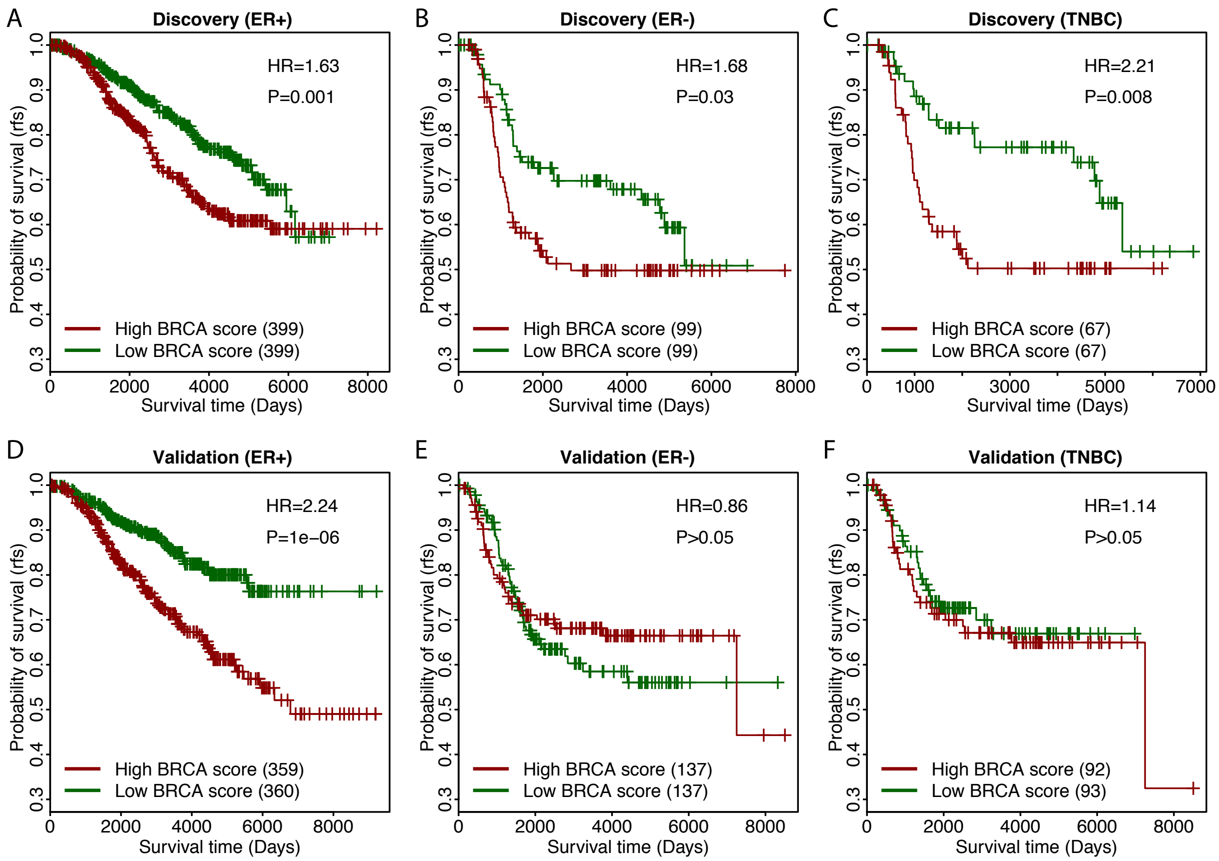

Supplementary Figure S4. BRCA scores correlate with patients' prognosis in additional datasets. Kaplan-Meier plots for the Ur-Rehman and Vijver datasets. Patients were divided into low and high BRCA score groups. Median of BRCA scores was applied as the cutoff. Red curves were patients with high BRCA scores and green curves were patients with low BRCA scores. Hazard ratio (HR) and log-rank test P-values were listed.

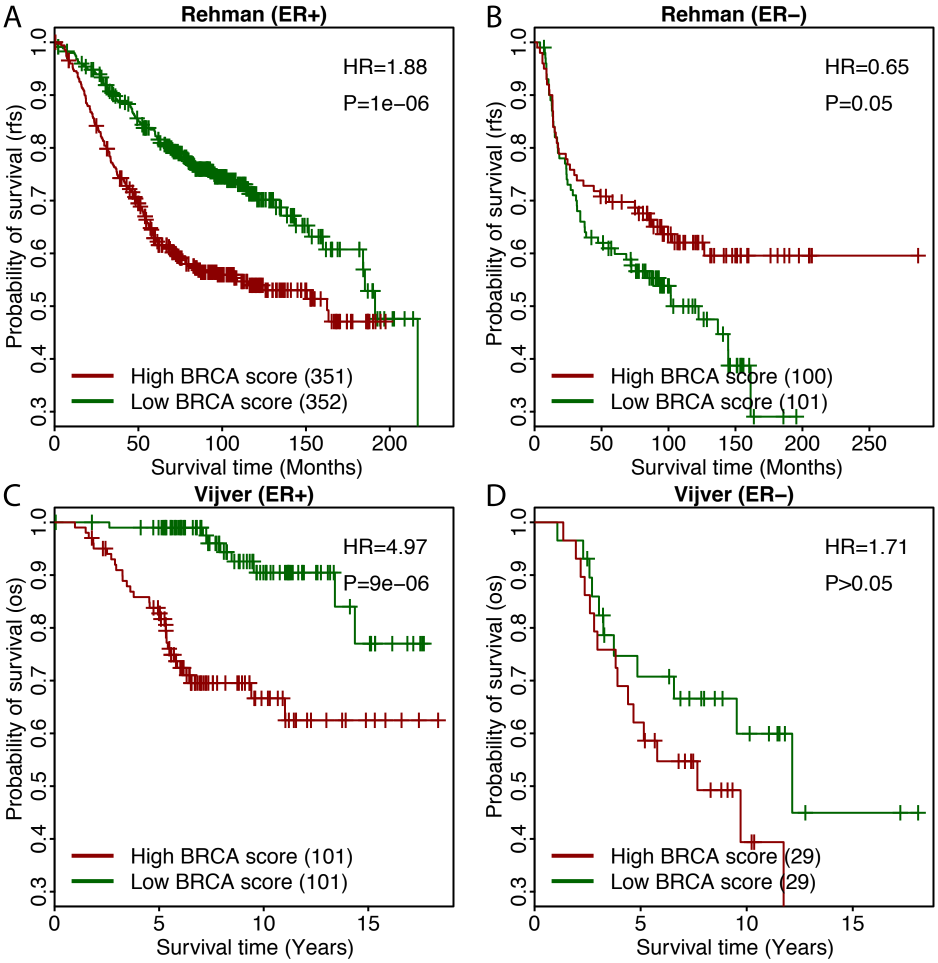

Supplementary Figure S5. BRCA scores correlate with neoadjuvant chemotherapy. We compared the BRCA scores for pathologic complete response (pCR) and residual disease (RD) patients with ER+, ER- and triple negative breast cancer (TNBC) samples. Mann–Whitney Wilcoxon test P-values were listed.

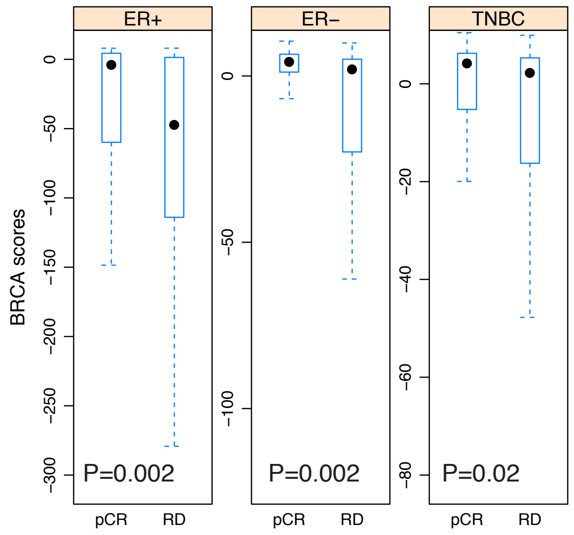

Supplementary Figure S6. Kaplan-Meier plots for the Yoshihara dataset. Patients were divided into non-BRCA-like and BRCA-like groups. Median of BRCA scores was applied as the cutoff. Red curves were BRCA-like patients with high BRCA scores and green curves were non-BRCA-like patients with low scores. Hazard ratio (HR) and log-rank test P-values were listed.

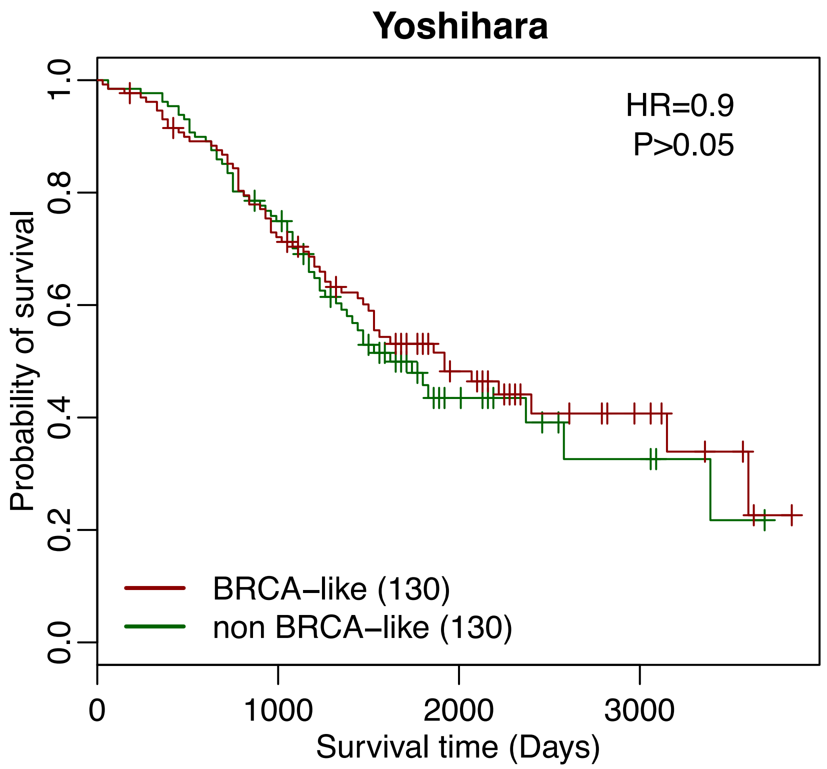

Supplementary Figure S7. BRCA scores correlate with BRCA1 and BRCA2 protein sequence. We mapped 13 and 14 mutations of BRCA1 and BRCA2 enrolled in TCGA dataset to the protein sequence of BRCA1 and BRCA2 and examined the BRCA score of corresponding samples. The red lines represent positive BRCA scores inferring HR pathway deficiency. In contrast, the green lines represent negative BRCA scores inferring higher HR pathway activity. Then length of line was proportional to the absolutely value of BRCA score. The colored squares represent different mutation types offered by TCGA.

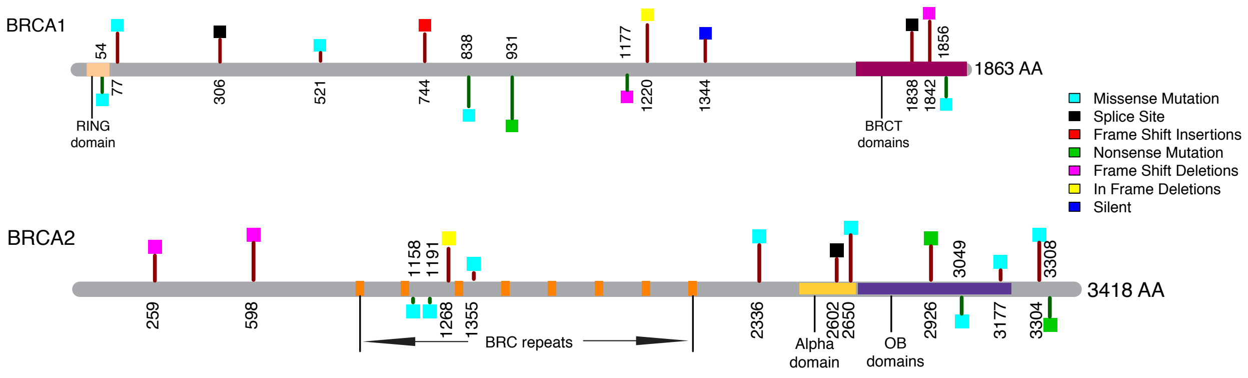

Supplementary Figure S8. BRCA scores are consistent using different BRCAness profiles. For each dataset, we calculated the sample-specific BRCA scores based on BRCA1-, BRCA2- and BRCA1/2-based BRCAness profiles, respectively. The BRCA scores showed highly consistency.

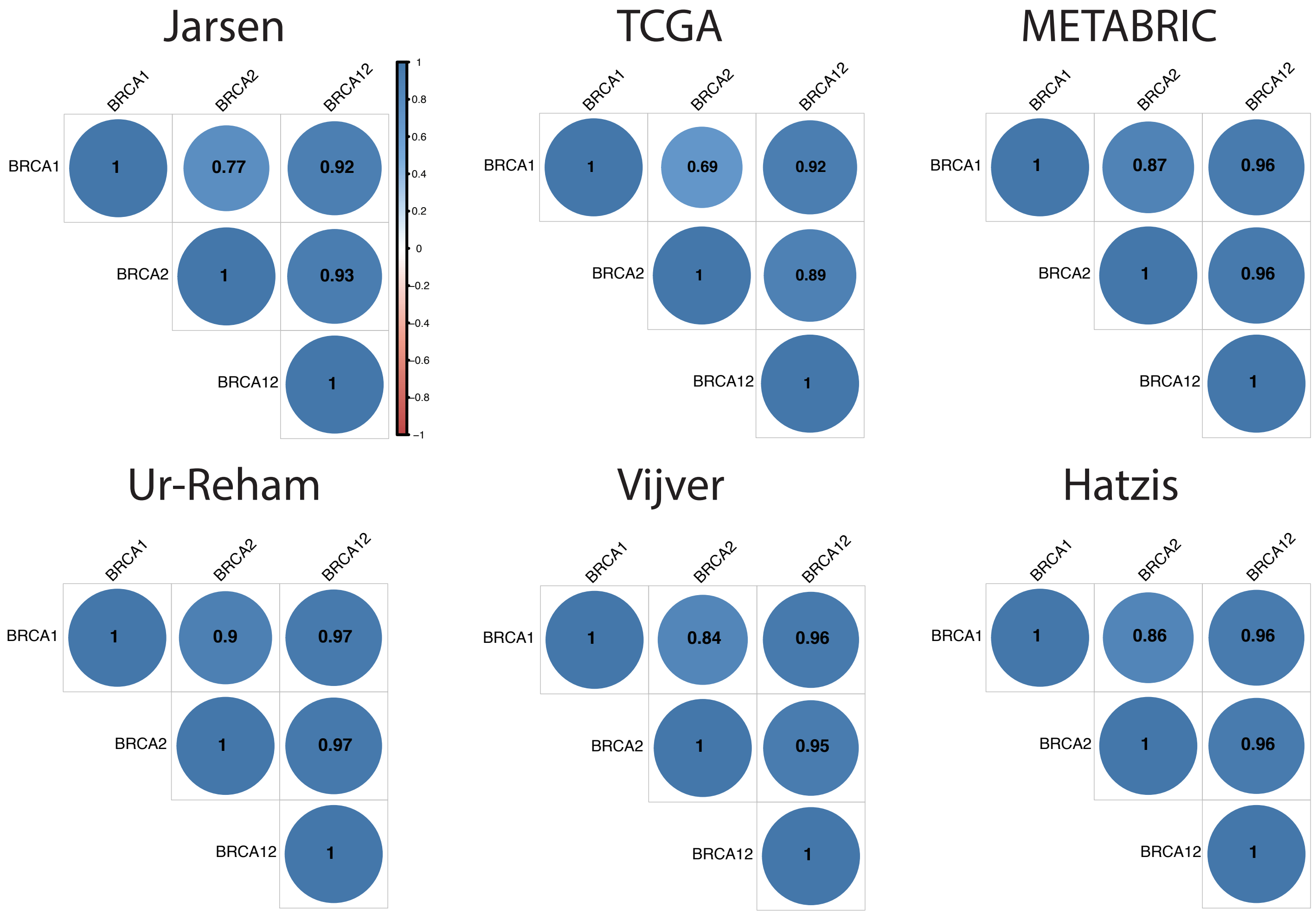

Supplementary Table S1. Pathways enriched in different gene groups. We investigated pathways enriched in genes up-regulated by BRCA1, BRCA2 and BRCA1/2-mutated samples but not for genes down-regulated in these patients.

Gene groups	REACTOME Pathways	#Gene in pathway	#Shared genes	P-value	Adjusted P-valu
	DNA_STRAND_ELONGATION	30	5	9.17E-05	2.78E-02
	DNA_REPLICATION	182	11	1.38E-04	2.78E-02
	CELL_CYCLE_MITOTIC	298	14	2.57E-04	2.78E-02
BRCA1_up	CELL_CYCLE	386	16	3.89E-04	2.78E-02
	PROCESSING_OF_CAPPED_INTRONLESS_PRE_MRNA	23	4	4.04E-04	2.78E-02
	TAK1_ACTIVATES_NFKB_BY_PHOSPHORYLATION_AND_ACTIVATION_OF_IKKS_COMPLEX	23	4	4.04E-04	2.78E-02
	EXTENSION_OF_TELOMERES	27	4	7.63E-04	4.50E-02
	DNA_REPLICATION	182	20	8.57E-12	3.54E-09
	MITOTIC_M_M_G1_PHASES	162	18	8.04E-11	1.66E-08
	CELL_CYCLE_MITOTIC	298	22	1.82E-09	2.50E-07
	CELL_CYCLE	386	22	1.89E-07	1.95E-05
	REGULATION_OF_MITOTIC_CELL_CYCLE	77	10	3.16E-07	2.61E-05
	SYNTHESIS_OF_DNA	84	10	7.22E-07	4.97E-05
	MITOTIC_PROMETAPHASE	86	10	9.00E-07	5.31E-05
	ORC1_REMOVAL_FROM_CHROMATIN	59	8	3.51E-06	1.67E-04
	S_PHASE	100	10	3.63E-06	1.67E-04
	CDK_MEDIATED_PHOSPHORYLATION_AND_REMOVAL_OF_CDC6	46	7	6.59E-06	2.72E-04
	HIV_INFECTION	191	13	9.81E-06	3.68E-04
	M_G1_TRANSITION	72	8	1.59E-05	5.47E-04
	CELL_CYCLE_CHECKPOINTS	105	9	3.89E-05	1.23E-03
	APC_C_CDH1_MEDIATED_DEGRADATION_OF_CDC20_AND_OTHER_APC_C_CDH1_TARGETED_PROTEINS_IN_LATE_MITOSIS_EARLY_G1	64	7	6.02E-05	1.78E-03
	APC_C_CDC20_MEDIATED_DEGRADATION_OF_MITOTIC_PROTEINS	65	7	6.66E-05	1.83E-03
	CDT1_ASSOCIATION_WITH_THE_CDC6_ORC_ORIGIN_COMPLEX	48	6	9.61E-05	2.48E-03
	HOST_INTERACTIONS_OF_HIV_FACTORS	120	9	1.10E-04	2.68E-03
	P53_DEPENDENT_G1_DNA_DAMAGE_RESPONSE	53	6	1.68E-04	3.51E-03
	SCFSKP2_MEDIATED_DEGRADATION_OF_P27_P21	53	6	1.68E-04	3.51E-03
	G1_S_TRANSITION	100	8	1.70E-04	3.51E-03
	ASSEMBLY_OF_THE_PRE_REPLICATIVE_COMPLEX	57	6	2.53E-04	4.97E-03
	ER_PHAGOSOME_PATHWAY	58	6	2.78E-04	5.22E-03
	ACTIVATION_OF_NF_KAPPAB_IN_B_CELLS	61	6	3.67E-04	6.36E-03
BRCA2_up	HIV_LIFE_CYCLE	112	8	3.69E-04	6.36E-03
	CYCLIN_E_ASSOCIATED_EVENTS_DURING_G1_S_TRANSITION_	62	6	4.01E-04	6.63E-03
	DOWNSTREAM_SIGNALING_EVENTS_OF_B_CELL_RECEPTOR_BCR	92	7	5.86E-04	9.32E-03
	CROSS_PRESENTATION_OF_SOLUBLE_EXOGENOUS_ANTIGENS_ENDOSOMES	46	5	7.20E-04	1.07E-02
	MITOTIC_G1_G1_S_PHASES	124	8	7.29E-04	1.07E-02
	AUTODEGRADATION_OF_THE_E3_UBIQUITIN_LIGASE_COP1	47	5	7.96E-04	1.13E-02
	ANTIGEN_PROCESSING_CROSS_PRESENTATION	71	6	8.32E-04	1.13E-02
	P53_INDEPENDENT_G1_S_DNA_DAMAGE_CHECKPOINT	48	5	8.77E-04	1.13E-02
	REGULATION_OF_ORNITHINE_DECARBOXYLASE_ODC	48	5	8.77E-04	1.13E-02
	MITOCHONDRIAL_PROTEIN_IMPORT	49	5	9.64E-04	1.14E-02
	SCF_BETA_TRCP_MEDIATED_DEGRADATION_OF_EMI1	49	5	9.64E-04	1.14E-02
	VIF_MEDIATED_DEGRADATION_OF_APOBEC3G	49	5	9.64E-04	1.14E-02
	DESTABILIZATION_OF_MRNA_BY_AUF1_HNRNP_D0	50	5	1.06E-03	1.21E-02
	P75_NTR_RECEPTOR_MEDIATED_SIGNALLING	79	6	1.46E-03	1.62E-02
	REGULATION_OF_APOPTOSIS	56	5	1.77E-03	1.87E-02
	AUTODEGRADATION_OF_CDH1_BY_CDH1_APC_C	56	5	1.77E-03	1.87E-02
	SIGNALING_BY_THE_B_CELL_RECEPTOR_BCR	121	7	2.89E-03	2.98E-02
	SIGNALING_BY_WNT	63	5	2.98E-03	3.00E-02
	CLASS_I_MHC_MEDIATED_ANTIGEN_PROCESSING_PRESENTATION	233	10	3.68E-03	3.62E-02
	ANTIGEN_PROCESSING_UBIQUITINATION_PROTEASOME_DEGRADATION	197	9	3.83E-03	3.68E-02
	MICRORNA_MIRNA_BIOGENESIS	21	3	4.04E-03	3.79E-02
	G0_AND_EARLY_G1	23	3	5.26E-03	4.72E-02
	ABORTIVE_ELONGATION_OF_HIV1_TRANSCRIPT_IN_THE_ABSENCE_OF_TAT	23	3	5.26E-03	4.72E-02
	CELL_CYCLE_MITOTIC	298	27	1.95E-13	8.05E-11
	DNA_REPLICATION	182	21	9.77E-13	2.02E-10
	CELL_CYCLE	386	29	2.75E-12	3.78E-10
	MITOTIC_M_M_G1_PHASES	162	19	9.06E-12	9.35E-10
	MITOTIC_PROMETAPHASE	86	11	9.44E-08	7.80E-06
	CELL_CYCLE_CHECKPOINTS	105	11	7.40E-07	5.09E-05
	REGULATION_OF_MITOTIC_CELL_CYCLE	77	9	3.05E-06	1.80E-04
	MITOTIC_G1_G1_S_PHASES	124	10	2.47E-05	1.21E-03
	G1_S_TRANSITION	100	9	2.63E-05	1.21E-03
	SYNTHESIS_OF_DNA	84	8	4.93E-05	2.03E-03
	E2F_MEDIATED_REGULATION_OF_DNA_REPLICATION	27	5	5.40E-05	2.03E-03
	APC_C_CDC20_MEDIATED_DEGRADATION_OF_MITOTIC_PROTEINS	65	7	6.66E-05	2.29E-03
	M_G1_TRANSITION	72	7	1.28E-04	4.08E-03
	MITOTIC_G2_G2_M_PHASES	74	7	1.53E-04	4.51E-03
BRCA1/2_up	S_PHASE	100	8	1.70E-04	4.67E-03
	G2_M_CHECKPOINTS	35	5	1.96E-04	5.06E-03
	GRB2_EVENTS_IN_ERBB2_SIGNALING	22	4	3.38E-04	8.21E-03
	ACTIVATION_OF_NF_KAPPAB_IN_B_CELLS	61	6	3.67E-04	8.35E-03
	TAK1_ACTIVATES_NFKB_BY_PHOSPHORYLATION_AND_ACTIVATION_OF_IKKS_COMPLEX	23	4	4.04E-04	8.35E-03
	KINESINS	23	4	4.04E-04	8.35E-03
	APC_C_CDH1_MEDIATED_DEGRADATION_OF_CDC20_AND_OTHER_APC_C_CDH1_TARGETED_PROTEINS_IN_LATE_MITOSIS_EARLY_G1	64	6	4.77E-04	9.38E-03
	DOWNSTREAM_SIGNALING_EVENTS_OF_B_CELL_RECEPTOR_BCR	92	7	5.86E-04	1.10E-02
	SIGNALING_BY_THE_B_CELL_RECEPTOR_BCR	121	8	6.20E-04	1.11E-02
	ADAPTIVE_IMMUNE_SYSTEM	508	18	1.08E-03	1.86E-02
	SIGNALING_BY_ILS	105	7	1.28E-03	2.12E-02
	ASSEMBLY_OF_THE_PRE_REPLICATIVE_COMPLEX	57	5	1.91E-03	3.04E-02
	ORC1_REMOVAL_FROM_CHROMATIN	59	5	2.23E-03	3.41E-02
	RNA_POL_II_TRANSCRIPTION	91	6	2.99E-03	4.41E-02
	SHC1_EVENTS_IN_ERBB4_SIGNALING	20	3	3.50E-03	4.99E-02

Supplementary Table S2. The result for multivariate Cox regression model. We constructed a multivariate Cox regression model to the METABRIC breast cancer dataset including both BRCA scores and clinical variables (*e.g.* age, ER status, Her2 status, stage and grade).

Varaible	Type	P value	HR (95% CI)
BRCA score high vs. low	Binary	3.0E-03	1.626 (1.180-2.241)
Age	Continuous	> 0.1	0.997 (0.986-1.009)
ER+ vs. ER-	Binary	> 0.1	0.831 (0.605-1.143)
HER2+ vs. HER2-	Binary	1.1E-03	1.750 (1.252-2.447)
Stage 2 vs. 1	Oridinal	2.0E-03	1.682 (1.209-2.340)
Stage 3 vs. 1	Oridinal	9.3E-13	4.692 (3.070-7.173)
Stage 4 vs. 1	Oridinal	1.6E-04	7.400 (2.621-20.898)
Grade 2 vs. 1	Oridinal	9.0E-02	2.085 (0.892-4.874)
Grade 3 vs. 1	Oridinal	1.7E-02	2.835 (1.209-6.649)

Supplementary Table S3. Summarization of datasets used in our study.

Cancer Type	Dataset	Accession ID	Briefly description
**Breast**	Larsen	GSE40115	55 familial (33 BRCA1 mutation and 22 BRCA2 mutation) and 128 sporadic breast tumor samples
	Lisowska	GSE50567	12 BRCA1- and 1 BRCA2-mutated hereditary breast tumors, 8 BRCAx (non-BRCA1/2 mutations) hereditary breast tumors, 14 sporadic breast cancer samples and 6 normal samples
	Foekens JA, M Martens JW, Smid M, Schutte M, Meijers-Heijboer H	GSE27830	155 familial primary breast cancer samples including 47 BRCA1-, 6 BRCA2-, 26 CHEK2- mutant samples and 76 samples without mutations in these three genes
	Waddell	GSE19177	19 BRCA1, 30 BRCA2 and 25 non-BRCA1/2 mutation familial breast cancer samples
	METABRIC	EGAS00000000083	1,992 primary breast cancer samples with comprehensive clinical information
	Ur-Rehman	GSE47561	1,170 samples integrated from existed breast cancer datasets
	Vijver	http://ccb.nki.nl/data/	295 breast cancer patients
	Hatzis	GSE25066	508 patients' response for neoadjuvant taxane-anthracycline chemotherapy
	TCGA	https://gdac.broadinstitute.org/	comprehensive data for BRCA samples
**Ovarian**	Jazaeri	GSE82007	18 *BRCA1*, 16 *BRCA2* germline mutant sample and 27 sporadic ovarian cancer samples
	Bonome	GSE26712	185 late-stage and high-grade patients
	Yoshihara	GSE32062	260 Japanese advanced-stage samples
	TCGA	https://gdac.broadinstitute.org/	comprehensive data for OV samples
